# Complement and diverse enrichment of antibodies in intraluminal thrombi from abdominal aortic aneurysms may contribute to increased inflammation

**DOI:** 10.1038/s41598-025-29506-0

**Published:** 2025-11-29

**Authors:** Vibeke Videm, Animesh Sharma, Torbjørn Dahl

**Affiliations:** 1 Department of Clinical and Molecular Medicine, NTNU – Norwegian University of Science and Technology, Trondheim, Norway; 2 Department of Immunology and Transfusion Medicine, St. Olavs University Hospital, Lab Center 3 east, Trondheim, 7006 Norway; 3 PROMEC Core Facility for Proteomics and Modomics, NTNU - Norwegian University of Science and Technology and the Central Norway Regional Health Authority, Trondheim, Norway; 4 Department of Surgery, St. Olavs University Hospital, Trondheim, Norway

**Keywords:** Abdominal aortic aneurysm, Intraluminal thrombus, Immune activation, Antibody formation, Proteomics, Biotechnology, Immunology, Diseases, Medical research, Pathogenesis

## Abstract

Abdominal aortic aneurysms (AAA) often contain an intraluminal thrombus (ILT) which may contribute to aneurysm growth and rupture, but the mechanisms are not well understood. We hypothesized that complement and antibodies play a central role in the immune reactions within ILT from AAA. We quantified pre-specified proteins from complement and other defense systems, including coagulation, fibrinolysis, neutrophils, relevant proteinases and inhibitors, as well as immunoglobulins in ILT (*n* = 7), using fresh thrombi for comparison (*n* = 7). Protein analysis was performed using liquid chromatography in tandem with mass spectrometry, permitting simultaneous protein detection with comparable quantitative results, and antibody diversity characterization. The results indicated complement activation (classical and lectin pathways), coagulation (contact pathway), and degranulation including proteinase release from neutrophils in the ILT, with protein enrichment compared to fresh thrombi. Inhibitors of these systems were also present. In the ILT, 151 different immunoglobulin heavy chain and 144 different light chain variable regions were detected. Corresponding numbers in the controls were 25 and 26. Immunohistochemistry confirmed terminal complement activation and substantial presence of IgG in the ILT, which also contained IgM and IgA. These results are suggestive of a complex immunological process with activation of many defense systems, and antibody enrichment in the ILT.

**Supplementary Information:**

The online version contains supplementary material available at 10.1038/s41598-025-29506-0.

## Introduction

An abdominal aortic aneurysm (AAA) is a localized dilatation usually in the infrarenal aorta, with a diameter of >=3 cm^1^. The prevalence is declining, and estimates have varied from < 1% to > 3% in men > 65 years depending on the population, and around 0.7% in women^2^. If undetected or untreated, aneurysmal growth may lead to aortic rupture, a condition carrying a lethality of ~ 80% when also considering patients who die before reaching hospital^3^. In approximately 70–80% of AAA patients, the aneurysm contains an intraluminal thrombus (ILT), which normally does not reduce blood flow^4^. In the area where the ILT is closest to the aortic wall, a layer of fluid of unknown significance is usually found^5^.

The most important risk factors for AAA development are male sex, family history and white ethnicity, as well as cigarette smoking^2,6^. The aortic wall in the aneurysm is characterized by apoptosis of smooth muscle cells and degeneration of the media. The mechanisms are not fully understood but involve activation and imbalance of matrix metalloproteinases (MMP), the renin-angiotensin system, intense inflammation involving many cell types and cytokines, and effects of reactive oxygen species and dysregulation of antioxidant systems. Sex hormones are also involved, but the data are conflicting^6^.

Several studies have indicated that not only the processes in the aortic wall itself are of importance, but that the ILT also is biologically active and may have a role in the growth and rupture of an AAA. The ILT has been viewed as a failed compensatory mechanism to protect against the further development of an AAA^7^. Two opposing mechanisms may be at play: the ILT may protect the underlying aortic wall against stress, but this may only be relevant for fresher ILT^5^ and may depend on the ILT size^8^. On the other hand, the wall below the ILT is thinner and shows more signs of tissue destruction and an active immune reaction, probably due to impaired oxygenation and release of inflammatory mediators and proteinases from the ILT^5,7,9^. These factors may increase the risk of rupture. Model and predictor studies for AAA growth have shown that the ILT thickness, placement, and geometry are important, indicating that there may be individual differences among patients^5,10^.

**Fig. 1:**
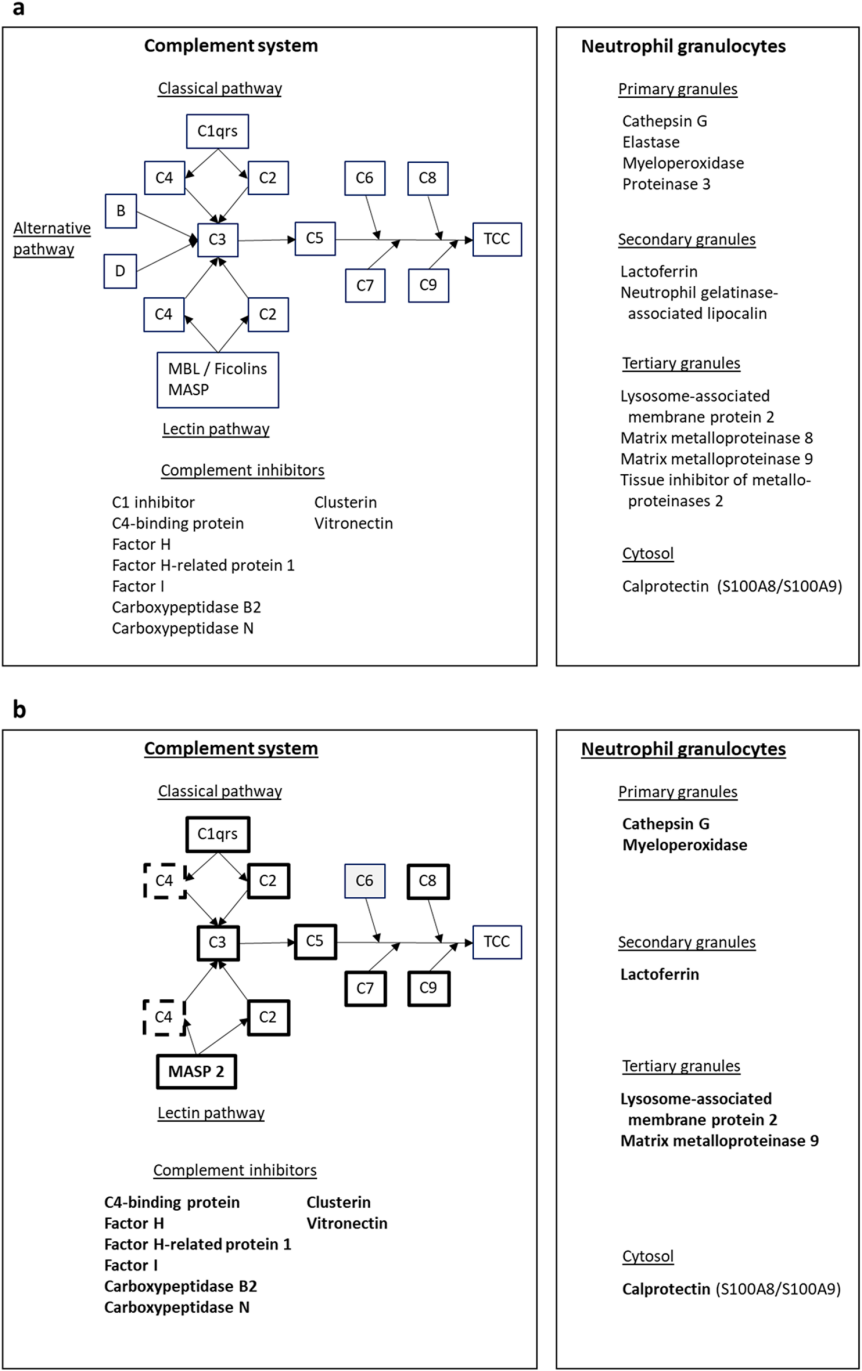
Investigated proteins from the complement system and neutrophil granulocytes. Panel **a**: Proteins pre-planned for investigation. The sequence of proteins in the complement system, as well as important inhibitors from plasma are indicated^11^. The biologically active activation products C3a and C5a are formed by enzymatic splitting of C3 and C5, respectively. The insert lists proteins released from neutrophils upon activation^19,20^. Panel **b** – Proteins found by proteomics. Most other proteins from panel a have been deleted, but the grey box indicates a missing complement factor that interrupts the sequence of terminal activation. Boxes with thick lines/bold text indicate proteins with significantly higher concentrations in the intraluminal thrombi than the control thrombi, apart from C2, where the concentration was significantly higher in the controls. Boxes with thick dotted lines indicate a protein with non-significant difference between intraluminal and control thrombi. MBL; mannose-binding lectin, MASP; MBL-associated serine proteinases, TCC; the terminal complement complex (C5b-C9).

To further characterize the biological effects of an ILT, several studies have been performed to investigate the presence and localization of proposed mediators. Most have focused on a small selection of relevant molecules, and few studies have focused on the complement system (schematically illustrated in Fig. 1a). Activation of the complement system with formation of various pro-inflammatory mediators including C3a, C5a, and the terminal complement complex (TCC) is essential for defense against infections. Complement also plays a role in removal of apoptotic cells and debris^11^. Furthermore, complement is interlinked with coagulation and fibrinolysis^11^. Activation products such as C3a and C5a may attract and activate immune cells including neutrophils^12^, which are an important source of proteinases in ILT^13^. Complement may also be activated by antibodies, but their role in ILT has not been thoroughly investigated.

We therefore hypothesized that complement and antibodies may play a central role in the immune reactions within ILT from AAA. The aim for the present study was to perform an exploratory, wide analysis covering relevant proteins from complement and other defense systems, including coagulation, fibrinolysis, neutrophils, relevant proteinases and inhibitors, as well as immunoglobulins in ILT and ILT fluid, using fresh thrombi for comparison. Based on the findings, we would then propose a model for how complement and antibodies participate in the defense system activation within the ILT from AAA. This information could be useful to better understand the potential effects of the ILT on the aortic wall during AAA formation and growth.

## Materials and methods

The study was approved by the Regional Committee for Medical and Health Research Ethics (REK Sør-øst A, Oslo, Norway, #2018/264, #7401) and followed the principles of the Helsinki declaration. All participants provided written informed consent before inclusion.

Power calculation was not performed because this was an exploratory study, and we found no previous relevant input data. To calculate a 95% confidence interval of the median result for continuous variables, minimum *n* = 6 observations are needed. We therefore considered that *n* = 7 would give a useful indication of whether complement and antibodies could play a central role in the immune reactions within ILT from AAA, even if there was a risk of false-negative findings.

**Fig. 2:**
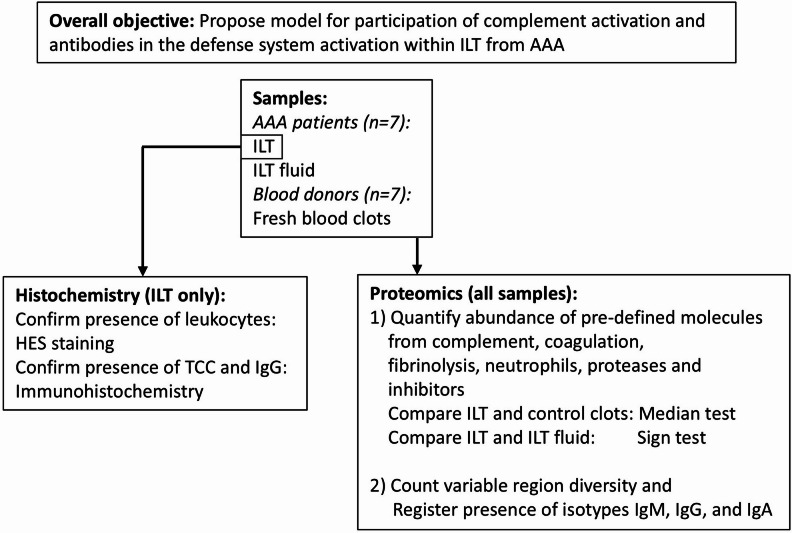
Objective and methods for the study. Samples and methods used to achieve the overall objective of the study. Details are given in the text. AAA; abdominal aortic aneurysm, HES; hematoxylin-erythrosine B-saffron, Ig; immunoglobulin, ILT; intra-luminal thrombus, TCC; terminal complement complex.

An overview of the study methodology is given in Fig. 2. Biomaterial from 7 patients (*n* = 5 men, *n* = 2 women), mean age 71 years (range: 57–78 years) undergoing open AAA surgery at St. Olavs University Hospital, Trondheim, Norway was included. No other clinical data than sex and age were collected. The biomaterial consisted of full-thickness samples from the ILT, and a sample from the fluid beneath the ILT. A part of the ILT was snap frozen in liquid nitrogen and later stored at −80 °C, whereas the ILT fluid was briefly kept at 4–6 °C and then stored in aliquots at −80 °C. For histochemistry, another part of the ILT was formalin-fixed and imbedded in paraffin.

Blood samples from 7 healthy volunteer blood donors drawn by the staff during routine blood donation at the hospital’s blood bank were also used for comparison to the ILT from the AAA. This material was chosen because blood donors represent a healthy population. The researchers were not given any information about the donors, in accordance with local regulations. Using tubes without anticoagulation, 6 mL samples were kept at room temperature for 2 h before centrifugation. Samples from the fresh thrombus were then transferred to clean tubes and stored at −80 °C.

**Fig. 3:**
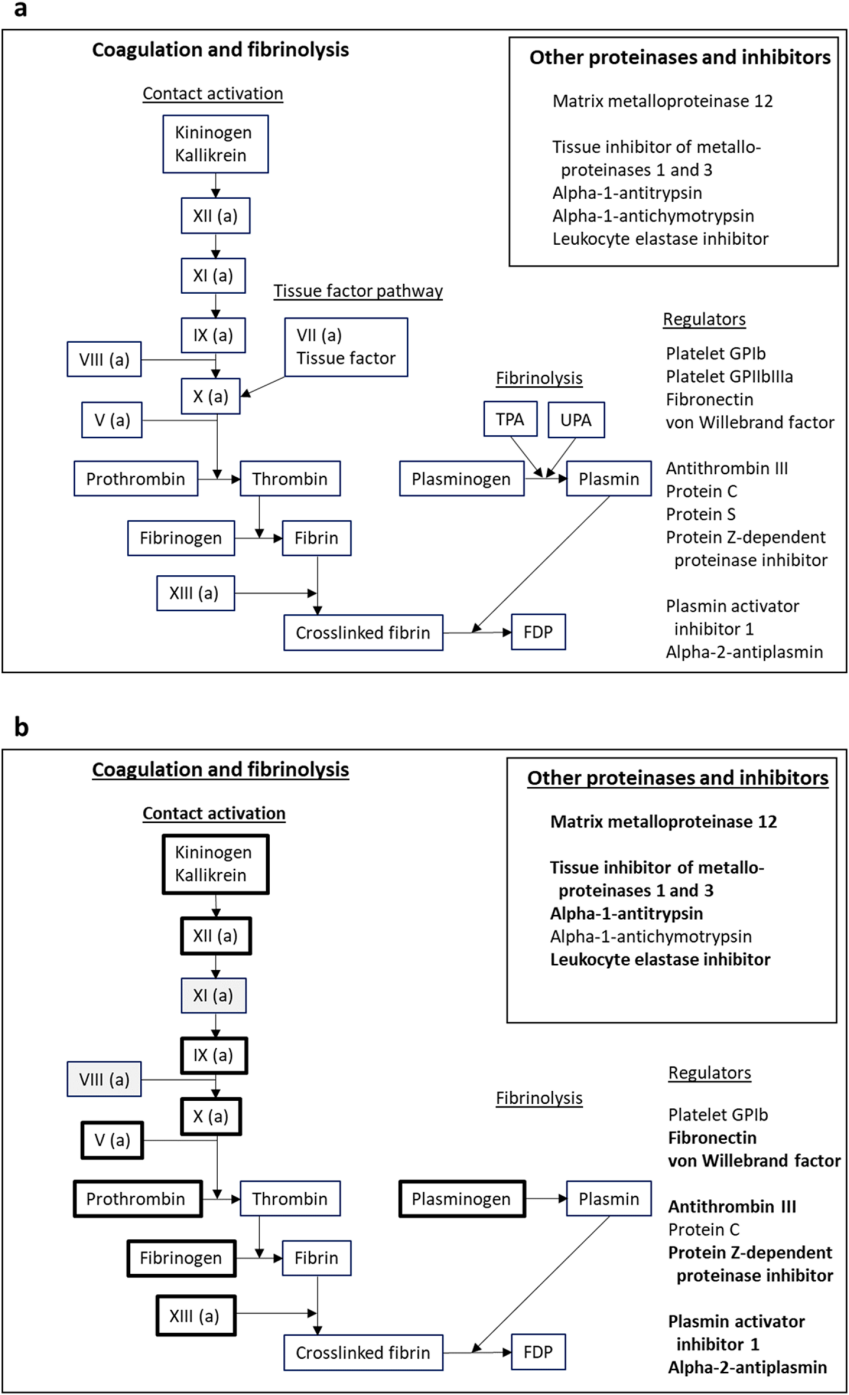
Investigated proteins from coagulation, fibrinolysis, and proteinases/inhibitors. Panel **a**: Proteins pre-planned for investigation. The sequence of proteins in coagulation and fibrinolysis^33^, as well as important regulators and accessory molecules from platelets and plasma are indicated^22,34^. The insert lists proteinases and protease inhibitors from macrophages and/or plasma^21,22^. Panel **b**: Proteins found by proteomics. Most other proteins from panel a have been deleted, but the grey boxes indicate missing proteins that interrupt the sequence of coagulation. Boxes with thick lines/bold text indicate proteins with significantly higher concentrations in the intraluminal thrombi than the control thrombi. FDP; fibrin degradation products, GP; glycoprotein, TPA ; tissue-type plasminogen activator, UPA; urokinase-type plasminogen activator.

To explore defense system involvement, the protocol pre-specified the proteins that would be investigated in the ILT, control thrombi, and ILT fluid using proteomics, as illustrated in Figs. 2a and 3a and detailed in Supplementary Table S1 online. The selection was based on subject knowledge and assessment of relevant literature. Data-dependent acquisition was used for this analysis because it permitted simultaneous detection of all the relevant proteins with comparable results, and detailed characterization of antibody diversity. Thus, the focus was on known proteins belonging to specific pre-defined pathways and cells. Detection of peptides from these proteins was taken to indicate that the protein was present in the sample.

Antibody molecules consist of 2 similar heavy chains and 2 similar light chains held together by disulfide bonds. Each antibody molecule has two identical antigen binding sites. These sites vary among different antibody molecules, resulting in antibody diversity. Both the heavy and light chains contain variable regions which in combination decide which antigen the antibody molecule can bind. Proteomics can be used to identify which such variable regions are present in the sample and thereby inform about the diversity of the investigated antibodies. However, adding up the relevant signals cannot be used to quantify total antibody abundance because the segments could be combined in many ways. Antibodies belong to different isotypes based on their constant regions, i.e. other parts of the molecule than the variable regions. IgG, IgM, and IgA are the most common isotypes in humans. IgM and IgA are multimeric, where several antibody molecules are held together by the so-called J chain. For our study, proteomics data achieved as described below were used to identify peptides from immunoglobulin heavy and light chain variable regions, heavy chain isotype-specific areas characterizing IgG, IgM, and IgA, and a peptide from the J chain, thus permitting characterization of antibody diversity.

### Proteomics analyses

Abundance of proteins from the defense systems was investigated in the samples using proteomics. Data-dependent acquisition was performed at the Proteomics and Modomics Experimental Core Facility (PROMEC) at NTNU – Norwegian University of Science and Technology, according to their standard operating procedures as previously published^14^. As starting material, a volume corresponding to approximately 100 mm^3^ or 100 µL of ILT, ILT fluid, or fresh control thrombus, respectively, was used. To each sample, 1mL of 0.1 M dithiothreitol, 30mM Tris pH 7.5 with complete protease inhibitor was added before homogenization using an Ultratorrax. Following addition of sodium dodecyl sulfate to a final concentration of 4%, the samples were heated at 70 °C for 15 min. Using Millipore Direct Detect (Merck, Darmstadt, Germany), the protein concentrations were measured before 200ug protein from each sample was precipitated using chloroform-methanol. This corrected for different protein concentrations in the starting material from each sample, for example due to different degrees of clot retraction in the fresh thrombi. Tryptic digestion of the pelleted proteins into peptides was done in 50mM ammonium bicarbonate using dithiothreitol/iodoacetamide as reducing/alkylating agent. After desalting, peptides were dried in a centrifuge concentrator (Eppendorf, Hamburg, Germany) before resuspension in 0.1% formic acid and analysis on a LC-MS/MS platform as described below.

The peptides from the digested samples were analyzed on a LC-MS/MS platform consisting of an Easy-nLC 1000 UHPLC system (Thermo Fisher Scientific) interfaced with an LTQ-Orbitrap Elite hybrid mass spectrometer (Thermo Fisher Scientific) via a nanospray ESI ion source (Proxeon, Odense, Denmark). Peptides were injected into a C-18 trap column (Acclaim PepMap100, 75 μm i. d. x 2 cm, C18, 3 μm, 100 Å, Thermo Fisher Scientific) and further separated on a C-18 analytical column (Acclaim PepMap100, 75 μm i. d. x 50 cm, C18, 2 μm, 100 Å, Thermo Fisher Scientific) using a multistep gradient with buffer A (0.1% formic acid) and buffer B (CH3CN, 0.1% formic acid) and a flow rate of 250 nL/min: 0–6% B in 5 min, 6–12% B in 39 min, 12–20% B in 80 min, 20–28% B in 31 min, 28–40% B in 4 min, 40–100% B in 1 min, 100% B for 9 min, 100-0% B in 1 min, and finally 10 min with 100% A. Eluted peptides were analyzed on the LTQ-Orbitrap Elite hybrid mass spectrometer operating in positive ion- and data-dependent acquisition mode using the following parameters: Electrospray voltage 1.9 kV, CID fragmentation with normalized collision energy 35, automatic gain control target value of 1E6 for Orbitrap MS and 1E3 for MS/MS scans. Each MS scan (m/z 300–1600) was acquired at a resolution of 120,000 FWHM, followed by 20 MS/MS scans triggered for intensities above 500, at a maximum ion injection time of 200 ms for MS and 120 ms for MS/MS scans.

Proteins were identified and quantified by processing the resulting MS data using Proteome Discoverer (PD, Thermo Scientific version 2.5, Waltham, MA, USA). Search parameters used: enzyme specified as trypsin with maximum two missed cleavages allowed; acetylation of protein N-terminal including loss-of-methionine, oxidation of methionine, and deamidation of asparagine/glutamine as dynamic post-translational modification while carbamidomethylation of cysteine as static; min-peptide length as 6; precursor mass-tolerance of 10 PPM while fragment mass-tolerance of 0.6 Dalton. PD’s node, Spectrum files RC, Minora, and Precursor-detector were set up to align/recalibrate, detect features and precursors, respectively. Further, the internal contaminants database was also queried along with the Human proteome including isoforms downloaded from PD’s knowledge base (Homo sapiens sp_tr_incl_isoforms TaxID = 9606_and_subtaxonomies) in November 2023 (v2023-11-08) using Sequest^15^ search engines available within the PD ecosystem. For downstream analysis of these peptide-spectra-matches, both protein and peptide identifications/peptide-spectra-matches, the false-discovery-rate was set to 1% as high and 5% as medium confidence; thus, only unique peptides with these confidence thresholds were used for final protein group identification and labelling of the level of confidence, respectively. Each protein group abundance was scaled on all averages and normalized by the total abundance of all identified peptides/peptide-spectra-matches at the false-discovery-rate mentioned earlier with the Precursor Ion Quantifier node of PD. This list was filtered to include only master proteins.

### Histochemistry

Histochemistry was used to confirm the presence and location of leukocytes, TCC, and IgG in the ILT. TCC and IgG were chosen because they have not been extensively studied in ILT previously, as opposed to molecules related to coagulation and fibrinolysis. Histochemistry was performed at the Cellular and Molecular Imaging Core Facility (CMIC), NTNU – Norwegian University of Science and Technology. The paraffin-embedded ILT were sectioned to 4 μm thickness. The sections were deparaffinized in Tissue Clear (3 × 5 min) and rehydrated in ethanol (100%, 96%, and 80%, 2 × 3 min in each) before rinsing in water (5 min). Heat-induced epitope retrieval to demask crosslinked protein epitopes was performed at pH = 6 (staining for IgG) or pH = 9 (staining for TCC) in PT-Link Target Retrieval Solution in a PT-link instrument (Dako Denmark, Glostrup, Denmark) before immunostaining using an autostainer (Dako Autostainer Plus) according to the manufacturer’s protocol. Briefly, endogenous peroxidase activity was blocked using Dako REAL Peroxidase Blocking Solution. Primary rabbit polyclonal antibodies were anti-human IgG (Dako A0423 diluted 1/1000, incubated 30 min at room temperature) and anti-C5b-9 (Thermo Fischer BS-2673R diluted 1/10, incubated overnight at 4 °C). The secondary antibody was HRP-anti-rabbit, and the substrate was DAB + Chromogen (both from REAL Envision Detection System K5007, Dako). Following counterstaining with hematoxylin and dehydration (Tissue-Tek Prisma Automated Slide Stainer, Sakura Finetek, Torrence, California, USA), the slides were mounted (Tissue-Tek Glasg2). Parallel sections were stained with hematoxylin-erythrosine B-saffron (HES), which stains nucleic acids dark blue/violet, cytoplasm, intercellular substances, and red blood cells pink/red, and collagen yellow/orange.

The slides were photographed in a VS-BX microscope (Olympus, Evident Europe GmbH, Hamburg, Germany) at 20X magnification using a VC50 camera (Olympus). Due to substantial variation within and among the ILT, no systematic scoring of immunohistochemistry staining intensity was possible.

### Statistics

The protein abundance data from the investigated defense systems were analyzed using Stata (v. 18.0, College Station, TX, USA) and are presented as median log2 normalized values with corresponding 25 and 75 percentiles. Two pre-planned comparisons were performed: Between the control clots and the ILT using the Median test, which is unpaired, and between the ILT and ILT fluid using the two-sided Sign test, which is paired, respectively. These methods have no assumptions about the distribution of data. Rank-based methods were employed because this would allow for inclusion of samples where the relevant proteins were not found. This was achieved by setting missing log2 normalized values to 1, which was very much lower than the lowest observed value (i.e., 9.11). Thus, the ranking order of the samples was preserved for the statistical tests. Only proteins that were found in at least 3 samples in at least 1 group were included in the statistical analysis. P-values were adjusted for multiple comparisons using the Benjamini-Hochberg method with a false discovery rate < 0.05 (https://tools.carbocation.com/FDR).

As explained above, data related to antibody diversity cannot be analyzed in the same way. Instead, we counted how many different variable regions were present in each sample, which isotype-specific regions were found, and whether the J-chain was present.

## Results

### Pre-specified proteins present in the samples

Figures 1b and 3b illustrate the pre-specified proteins that were found in the samples. There was indication of complement activation by the classical and lectin pathways, but not by the alternative pathway (Fig. 1b). Factor C6 was not found. The employed method could not identify peptides specific for the terminal complement complex, which is a multimer consisting of the C5b fragment from C5 and the molecules C6, C7, C8, and C9. Immunohistochemistry confirmed that TCC was present in the ILT, as further described below. Most of the pre-specified inhibitors of complement were also increased in the ILT, including factor H-related protein 1, clusterin, and vitronectin that inhibit assembly of the terminal complement complex^11^.

There was indication of activation of the contact pathway of coagulation (Fig. 3b), even if factors XI and VIII were not found. Neither tissue factor nor factor VII, constituting the tissue factor pathway, were found. The employed method could not differentiate between prothrombin and thrombin, fibrinogen and fibrin, or plasminogen and plasmin, respectively, because the identified peptide sequences were similar for both forms. However, previous studies have confirmed fibrin deposition and fibrinolysis in ILT from AAA. Fibrin, fibrin degradation products (an indirect indication of fibrinolysis), and plasmin were demonstrated in studies using Western blotting^16,17^, and histology using Masson’s trichrome staining documented both fibrin and degraded fibrin^18^. Several of the pre-specified regulators of coagulation and thrombus formation were also present (Fig. 3b).

At least one degranulation product from each of the primary (cathepsin G, myeloperoxidase), secondary (lactoferrin), and tertiary granules (lysosome-associated membrane protein 2 – LAMP2, matrix metalloproteinase 9 – MMP9) of granulocytes were found, as well as calprotectin, which is released from the cytosol upon activation (Fig. 1b)^19,20^. In addition, MMP12 and several proteinase inhibitors from platelets, macrophages, or plasma were identified (insert in Fig. 3b)^21,22^.

The median log2 normalized values of the identified proteins and results from the statistical comparisons are given in Tables 1 and 2, and 3. There was strong evidence of protein enrichment in the ILT compared to the fresh thrombi for all the investigated defense systems, both with respect to the number of thrombi in which the proteins were present and their concentrations. There were no significant differences in protein concentrations between the ILT and the ILT fluid.

**Table 1:** Complement system - median log2 normalized values for observed proteins.

Protein	Controls (C)	C number^1^	Intraluminal thrombus (ILT)	ILT number^1^	Fluid (F)	F number^1^	*P*-value^2^ C vs. ILT	*P*-value^2^ ILT vs. F
C1q alpha	1 (1, 1)	0	20.15 (18.70, 20.31)	6	19.71 (1, 21.06)	5	0.002	1.00
C1q beta	1 (1, 1)	0	19.51 (18.75, 19.67)	7	19.60 (1, 20.37)	5	< 0.001	1.00
C1q c	20.94 (1, 21.18)	4	22.09 (20.94, 22.30)	7	21.83 (20.95, 22.75)	7	0.11	1.00
C1r	1 (1, 1)	0	20.68 (19.37, 21.74)	7	21.33 (16.46, 21.99)	6	< 0.001	1.00
C1s	1 (1, 16.65)	3	18.84 (17.56, 19.60)	7	20.23 (17.53, 20.72)	6	0.009	0.64
C2	23.00 (22.39, 24.08)	7	22.15 (21.57, 22.37)	7	22.87 (22.45, 22.96)	7	0.009	0.070
C3 (partial sequence)	1 (1, 1)	0	17.60 (1, 17.93)	5	19.10 (17.53, 19.20)	6	0.008	0.52
C3 (complete sequence)	19.65 (18.81, 20.03)	7	26.43 (25.29, 26.56)	7	26.66 (24.09, 26.81)	7	< 0.001	0.64
C4 alpha	1 (1, 1)	0	1 (1, 17.32)	3	17.62 (1, 19.69)	5	0.055	0.23
C4 beta	1 (1, 1)	0	1 (1, 16.46)	3	16.45 (1, 17.45)	4	0.055	0.64
C5	1 (1, 16.87)	3	22.63 (22.58, 23.41)	7	22.84 (19.31, 23.92)	7	< 0.001	1.00
C7	1 (1, 1)	0	20.03 (19.18, 20.72)	7	19.93 (1, 21.17)	5	< 0.001	1.00
C8 alpha	1 (1, 1)	0	15.77 (1, 18.01)	4	1 (1, 18.67)	2	0.020	1.00
C8 beta	1 (1, 1)	1	20.70 (19.66, 21.05)	7	19.84 (17.43, 21.20)	7	< 0.001	0.64
C8 gamma	1 (1, 1)	0	20.31 (19.53, 20.72)	7	20.06 (1, 20.91)	5	< 0.001	1.00
C9	16.97 (1, 18.77)	5	23.43 (23.24, 23.83)	7	22.46 (19.69, 23.93)	7	< 0.001	0.64
MBL-associated serine protease 2	1 (1, 1)	0	17.06 (15.59, 18.04)	6	17.16 (1, 17.44)	5	0.002	1.00
C4b-binding protein alpha	1 (1, 1)	1	22.63 (22.59, 23.64)	7	23.81 (16.83, 25.13)	7	< 0.001	0.64
C4b-binding protein beta	1 (1, 1)	0	17.81 (17.40, 18.91)	7	19.27 (1, 20.42)	5	< 0.001	0.64
Factor H	1 (1, 1)	0	20.87 (20.31, 21.38)	7	21.49 (16.79, 22.12)	7	< 0.001	0.64
Factor H-related protein 1	1 (1, 1)	0	18.48 (1, 18.92)	5	1, (1, 17.23)	2	0.008	0.23
Factor I	1 (1, 1)	0	20.06 (19.09, 20.58)	7	20.90 (1, 21.02)	5	< 0.001	0.64
Carboxypeptidase B2	1 (1, 1)	0	21.10 (20.40, 21.37)	7	17.84 (1, 18.88)	6	< 0.001	0.070
Carboxypeptidase N	1 (1, 1)	0	16.72 (15.58, 17.12)	6	17.99 (1, 18.54)	5	0.002	0.52
Clusterin	19.33 (18.81, 19.75)	7	24.66 (24.20, 24.94)	7	22.44 (21.59, 23.53)	7	< 0.001	0.070
Vitronectin	10.93 (1, 16.22)	5	25.20 (24.46, 25.56)	7	23.12 (18.86, 23.83)	7	< 0.001	0.070

^1^ Number of samples in group where the protein was detected.

^2^ Benjamini-Hochberg-adjusted p-value using false discovery rate < 0.05.

**Table 2:** Coagulation and fibrinolysis - median log2 normalized values for observed proteins.

Protein	Controls (C)	C number^1^	Intraluminal thrombus (ILT)	ILT number^1^	Fluid (F)	F number^1^	*P*-value^2^ C vs. ILT	*P*-value^2^ ILT vs. F
Kininogen 1	1 (1, 15.58)	2	22.22 (22.12, 22.71)	7	23.32 (20.91, 23.49)	7	< 0.001	0.64
Plasma kallikrein	18.32 (1, 18.57)	5	20.08 (19.40, 20.83)	7	20.73 (18.41, 21.00)	7	0.009	0.39
Factor V	1 (1, 1)	1	19.44 (17.41, 19.57)	6	18.24 (17.16, 18.93)	6	0.009	0.82
Factor IX	1 (1, 1)	0	19.85 (19.40 20.85)	7	17.57 (1, 19.20)	5	< 0.001	0.070
Factor X	1 (1, 1)	0	19.19 (18.57, 19.62)	7	17.49 (1, 18.40)	5	< 0.001	0.39
Factor XII	1 (1, 1)	0	16.99 (1, 18.17)	5	18.53 (1, 18.99)	5	0.008	0.52
Factor XIII alpha	1 (1, 1)	1	20.11 (19.72, 20.45)	7	16.14 (1, 16.96)	5	< 0.001	0.070
Factor XIII beta	1 (1, 1)	1	19.03 (17.42, 19.28)	6	17.67 (16.09, 18.25)	6	0.009	0.070
Prothrombin	15.53 (1, 17.05)	5	24.50 (23.00, 24.73)	7	23.31 (20.09, 23.82)	7	< 0.001	0.64
Fibrinogen alpha	22.37 (22.14, 22.87)	7	28.86 (28.14, 28.99)	7	26.38 (23.05, 26.98)	7	< 0.001	0.070
Fibrinogen gamma	22.33 (21.85, 22.64)	7	29.39 (28.50, 29.68)	7	26.93 (23.02, 27.42)	7	< 0.001	0.070
Plasminogen	1 (1, 1)	1	25.47 (24.05, 25.49)	7	22.88 (17.16, 23.59)	7	< 0.001	0.070
Platelet glycoprotein Ib alpha	1 (1, 17.22)	2	17.01 (10.30, 17.52)	6	17.68 (17.57, 19.03)	7	0.11	0.070
Platelet glycoprotein Ib beta	1 (1, 1)	0	17.35 (1, 19.17)	5	1 (1, 15.18)	2	0.008	0.52
Fibronectin (partial sequence)	1 (1, 1)	0	17.91 (15.16, 19.86)	6	16.98 (1, 17.33)	4	0.002	1.00
Fibronectin (complete sequence)	16.69 (16.25, 17.20)	7	23.87 (23.56, 24.26)	7	24.42 (20.07, 24.71)	7	< 0.001	0.64
von Willebrand factor	15.33 (1, 15.69)	5	16.53 (15.94, 17.16)	6	19.90 (16.45, 20.59)	6	0.009	0.52
Antithrombin III	20.05 (19.74, 20.17)	7	21.60 (21.54, 21.76)	7	22.32 (20.71, 22.70)	7	< 0.001	0.64
Protein C	1 (1, 1)	0	1 (1, 15.65)	2	15.87 (1, 16.15)	4	0.13	0.39
Protein Z-dependent protease inhibitor	1 (1, 1)	0	17.25 (1, 18.50)	4	1 (1, 15.41)	2	0.020	0.64
Plasminogen activator inhibitor 1	1 (1, 1)	0	14.96 (1, 16.05)	5	15.53 (1, 16.82)	4	0.008	1.00
Alpha-2-antiplasmin	1 (1, 1)	0	22.83 (21.32, 22.88)	7	21.74 (15.07, 21.94)	7	< 0.001	0.070

^1^ Number of samples in group where the protein was detected.

^2^ Benjamini-Hochberg-adjusted p-value using false discovery rate < 0.05.

**Table 3:** – Neutrophil granulocytes, other proteinases and inhibitors - median log2 normalized values for observed proteins.

Protein	Controls (C)	C number^1^	Intraluminal thrombus (ILT)	ILT number^1^	Fluid (F)	F number^1^	*P*-value^2^ C vs. ILT	*P*-value^2^ ILT vs. F
Neutrophil granulocytes								
Cathepsin G	14.92 (1, 15.71)	4	20.22 (19.32, 20.52)	7	16.85 (1, 18.30)	5	0.008	0.070
Myeloperoxidase	15.85 (1, 16.64)	4	19.05 (18.75, 21.09)	6	18.03 (17.44, 19.61)	7	0.009	1.00
Lactoferrin	1 (1, 15.77)	2	20.45 (18.86, 20.94)	7	19.96 (19.62, 20.23)	7	< 0.001	0.64
Lysosome-associated membrane glycoprotein 2	1 (1, 1)	0	15.98 (1, 16.84)	4	15.54 (1, 15.92)	4	0.020	1.00
Matrix metalloproteinase 9	1 (1, 1)	0	15.41 (1, 16.47)	4	1	1	0.020	0.84
Protein S100-A8	18.14 (17.57, 19.13)	7	21.47 (20.65, 22.29)	7	21.96 (20.20, 23.14)	7	0.009	0.64
Protein S100-A9	18.37 (17.21, 18.75)	6	20.00 (18.83, 21.51)	7	20.52 (20.15, 22.02)	7	0.009	0.64
**Other proteinases and inhibitors**								
Matrix metalloproteinase 12	1 (1, 1)	0	17.96 (13.84, 20.63)	6	15.51 (1, 18.81)	4	0.002	0.91
Tissue inhibitor of metalloproteinases 1	1 (1, 1)	0	19.31 (18.02, 19.61)	7	18.57 (1, 19.53)	5	< 0.001	1.00
Tissue inhibitor of metalloproteinases 3	1 (1, 1)	0	16.01 (1, 19.29)	5	14.76 (1, 16.02)	4	0.008	1.00
Alpha-1-antitrypsin	1 (1, 16.53)	2	17.87 (1, 19.88)	4	19.97 (1, 20.48)	5	0.28	0.52
Alpha-1-antichymotrypsin	1 (1, 1)	0	23.14 (22.92, 23.69)	7	24.03 (22.09, 24.25)	7	< 0.001	0.64
Leukocyte elastase inhibitor	1 (1, 1)	1	15.35 (14.08, 17.59)	6	1	1	0.009	0.13

^1^ Number of samples in group where the protein was detected.

^2^ Benjamini-Hochberg-adjusted p-value using false discovery rate < 0.05.

### Immunoglobulin diversity in the samples

Immunoglobulins were present in all samples, but with different variable region diversity in the three groups. In the control thrombi, 25 different heavy chain and 26 different light chain variable regions were detected. In the ILT, the corresponding numbers were 151 and 144, and in the ILT fluid, they were 186 and 184, respectively. Isotype markers identified only IgG in the control thrombi, and IgM, IgG (IgG3 and IgG4), IgA, as well as the J chain in the ILT and ILT fluid. These results are suggestive of an active immunological process with antibody enrichment in the ILT and ILT fluid.

### Histochemistry

**Fig. 4:**
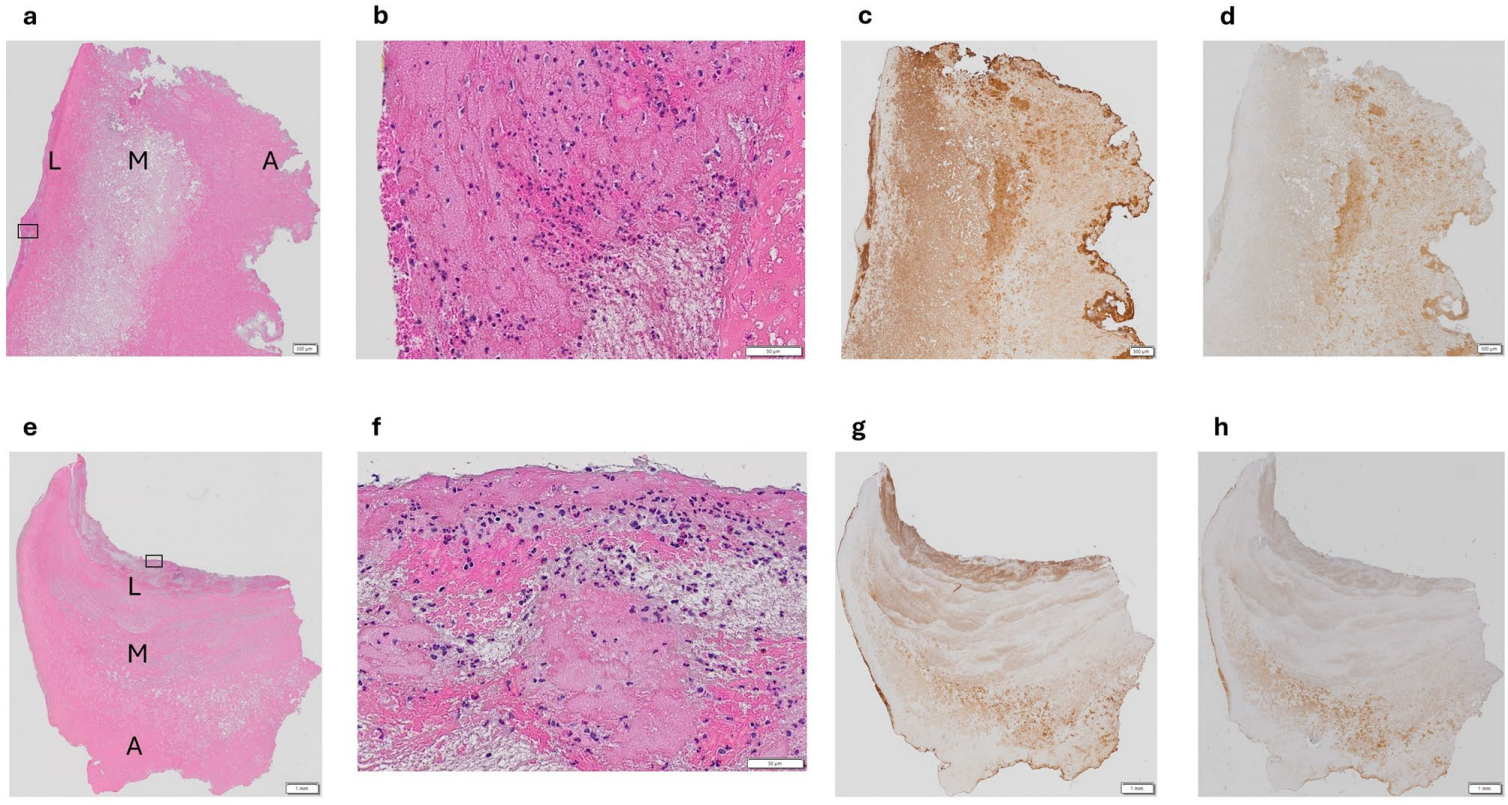
Histochemistry of intraluminal thrombi. Representative images from patient 1 (upper row) and patient 2 (lower row). Magnification: scalebar box width corresponds to 500 micrometers in a, c, d, e, g, and h, and 50 micrometers in b and f. Panels **a** and **e**: Overview of hematoxylin-erythrosine B-saffron-stained thrombi showing mostly erythrosine B-stained layers with different density. L = luminal layer, M = medial layer, A = abluminal layer. Panels **b** and **f**: Magnification from the luminal layer indicated by boxes in panels a and f, showing many neutrophil granulocytes, monocytes, and lymphocytes (nuclei stained purple). Panels **c** and **g**: Immunostaining for immunoglobulin G (brown), showing abundant presence in several layers. Panels **d** and **h**: Immunostaining for the terminal complement complex (brown), showing presence mostly in the middle and abluminal layers.

The HES-stained ILT sections showed that the thrombi mostly consisted of erythrosine B-stained layers with different density (representative examples in Fig. 4). The luminal parts contained many neutrophil granulocytes, monocytes, and lymphocytes (Fig. 4, panels b and f), whereas leukocytes were generally not present in the medial and abluminal parts. Immunohistochemistry showed rich staining for IgG with varying location among the ILT (Fig. 4, panels c and g), and staining for TCC mostly in the medial and abluminal layers (Fig. 4, panels d and h).

## Discussion

In the present study comparing ILT from AAA to fresh thrombi, we found evidence of complement activation by the classical and lectin pathways, and presence of many complement inhibitors. There was indication of activation of the contact pathway of coagulation in the ILT, and also fibrinolysis. Many regulators of coagulation were also present. Important degranulation products from neutrophils were detected. MMP12 and several proteinase inhibitors from platelets, macrophages, and plasma were identified. There were no significant differences in concentrations of these proteins between the ILT and ILT fluid, which probably has not been investigated before. On the other hand, immunoglobulin variable region diversity was substantially greater in ILT than in control thrombi, and IgM, IgG, IgA, and the J chain were found in the ILT and ILT fluid. Histochemistry confirmed the presence of leukocytes including neutrophils in the luminal parts of the ILT, as well as IgG in different parts and TCC mostly in the medial and abluminal parts.

The results support an active immunological process with activation of many defense systems and antibody enrichment in the ILT and ILT fluid. The presence of proinflammatory molecules as well as relevant inhibitors support the notion that the defense systems participate in a complex, regulated process in the ILT. Even if C6 was not present, immunohistochemistry confirmed that the terminal complement complex was formed. A previous study found colocalization of C5b-9 and C3 in ILT, which also supports activation of the terminal pathway^23^. C3a and C5a are probably the most relevant complement effector molecules in the present setting.

**Fig. 5:**
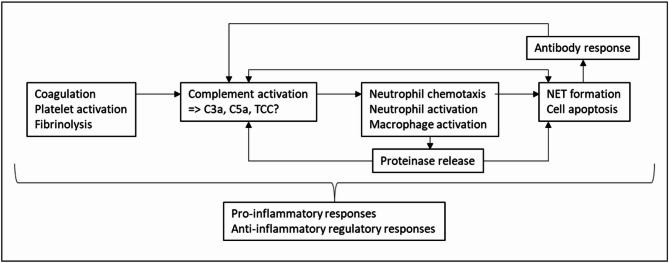
Proposed relationships among defense systems in the intraluminal thrombus. Complement activation follows from the ongoing activation of coagulation, platelets, and fibrinolysis. Complement activation products such as C3a and C5a, potentially also the terminal complement complex (TCC), lead to neutrophil chemotaxis and accumulation in the ILT, as well as neutrophil activation with proteinase release. Apoptosis of cells including neutrophils and macrophages induced by various mechanisms and proteinase digestion of ILT constituents lead to formation of apoptotic bodies and debris that may further activate complement, as well as act as antigens for an antibody response taking place in regional lymph nodes or tertiary lymphoid organs in the adventitia of the aortic wall of the aneurysm (not illustrated). Accumulating antibodies in the ILT contribute to further complement activation. The pro-inflammatory responses also evoke anti-inflammatory regulatory mechanisms. C3a; complement fragment C3a, C5a; complement fragment C5a, NET; neutrophil extracellular traps, TCC; terminal complement complex,.

Coagulation and fibrinolysis are linked to activation of the complement system, e.g. by activated factor IX and X, thrombin, or plasmin^11^, which were present in the ILT in our study. Additional complement activation could play an important role for the further cycle of processes in the ILT, for which a proposed scheme is shown in Fig. 5.

The complement activation products C3a and C5a are important chemotactic factors and activators of neutrophils. Chemotaxis is also induced by RANTES (Regulated on Activation, Normal T cell Expressed and Secreted) and interleukin-8 released from platelets in the ILT^24^. Several previous studies have also shown that neutrophils are mostly present in the luminal part of the ILT, where activation and degranulation leads to release of important proteinases^25^. Some of the detected proteins may also be released from other cell types. Our study showed that proteinases from the three main neutrophil granule types as well as proteins from the cytoplasm were present in the ILT, and the aggregate results strongly suggest neutrophil activation. Upon activation, neutrophils may also form extracellular traps (NET) when they extrude DNA which has bound nuclear and cytoplasmic proteins, whereafter the cell dies after a few minutes^26^. Neutrophil elastase and probably other neutrophil proteinases are involved in NET formation (NETosis). NET may also interact with platelets and enhance fibrin formation.

A previous morphological study found many neutrophils undergoing apoptosis in ILT^18^. Complement activation products and proteinases are probable causes of apoptosis of neutrophils and macrophages in the ILT. Apoptosis, NETosis, and formation of debris will expose antigens that may elicit an antibody response. We found enrichment of antibody diversity within the ILT, indicated by the increasing number of different heavy and light chain variable regions. Immunohistochemistry confirmed substantial presence of IgG, the most common isotype during long-term responses.

During formation of receptors in developing B cells in the bone marrow, gene segments coding for the antigen-binding sites of the heavy and light chains constituting the receptor are recombined and spliced by inaccurate mechanisms. The ensuing receptors are surface-bound antibody molecules, equal on each mature (naïve) cell, but with a large receptor diversity among different B cells. During an immune response, a naïve B cell with a receptor that binds an antigen undergoes maturation to a clone of plasma cells producing antibody against the antigen, which is then secreted from the cell instead of being expressed on the surface. Such B cell development into plasma cells does not occur in the ILT itself, but in lymphoid tissue where other necessary cells like T follicular helper cells are also present. In AAA patients, this could be local lymph nodes. Recent data also show that the adventitia of the aorta in the aneurysm contains tertiary lymphoid organs with B cells and active release of antibodies, which is also a probable location for this part of the ILT-related immune response^27^. Our findings support that many different naïve B cells are activated by antigens in the ILT, resulting in secretion of antibodies coded by different variable region gene segments. However, we cannot exclude that the activating antigens may also be present in other locations, e.g. the aortic wall. In any case, the antibodies that are released from the lymphoid organs into the circulation are then enriched in the ILT from plasma because they bind to the relevant antigens. Some antibody was also found in the fresh control thrombi. These may have been trapped from plasma during coagulation. This is supported by the fact that the only isotype present was IgG, which is the most abundant plasma isotype.

It is impossible to identify the specific antigens based only on identification of the variable regions of the antibodies, so our study cannot answer which antigens are involved in the responses within an ILT. However, it is probable that many of these antibodies may be so-called natural antibodies, which are often directed against apoptotic cells and other self-antigens^28^. Natural antibodies are often IgM but may also be IgG or IgA, especially when there is ongoing immunological activation which induces isotype change from IgM to IgG. To support this notion, a previous study found complement-activating antibodies against different fibrinogen chains in serum from AAA patients, which also bound to tissue from their aneurysms^29^. We may speculate that strong, long-lasting expression of fibrinogen and/or fibrin as well as other self-antigens in the ILT recognized by natural antibodies may predispose to increased antibody production, which in turn contributes to AAA growth when circulating antibodies later bind to self-antigens in the aortic wall and elicit further inflammation there.

Such natural antibodies may also lead to further complement activation in the ILT (Fig. 3), contributing to a perpetuated inflammatory response. This is supported by the finding of TCC by immunohistochemistry in the ILT. IgM, IgG1, and IgG3 are potent activators of complement via the classical pathway^11,30^. IgG4 was found in all the ILT and ILT fluids in the present study. IgG4 is not complement-activating and acts as a negative regulator of inflammation in many settings^31^. Thus, the presence of IgG4 does not mean that the investigated patients had inflammatory aneurisms due to IgG4-related disease, which may sometimes manifest with retroperitoneal fibrosis.

We found similar concentrations of proteins in ILT and ILT fluid. Previous studies have shown that fluid and macromolecules pass freely from the luminal to the abluminal surface by centrifugal forces through a network of channels or “canaliculi” that traverse the ILT^13,16^, and therefore come into close contact with the wall of the AAA. Proteinases in the ILT may be partly bound to inhibitors^25^. Our study demonstrated that not only proteinase inhibitors, but many regulators or inhibitors of complement, coagulation, and fibrinolysis were present in the ILT and ILT fluid. It does not give information about the level of activity of the various molecules. However, the binding of TIMP1 to proteinases is reversible, and it also acts as a proinflammatory cytokine via other mechanisms^32^. We may speculate that even if some mediators are bound to inhibitors and therefore are not fully active, some remaining activity in the combined “soup” of bioactive molecules in close proximity to the aortic wall may still have detrimental effects.

The study has some limitations. Due to practical and financial restraints, only a small number of samples could be analyzed in this exploratory study. The biggest concern with small studies is the risk of false-negative conclusions. The findings confirm that the present study was large enough to shed light on the research question. To investigate whether the inflammatory responses in ILT fall into identifiable groups when comparing different patients, a very much larger study would be needed. The patients were not pre-selected, but we cannot exclude that they may not have been representative of the population with AAA. All participants had advanced disease, and the findings may not be relevant for earlier phases in AAA development. We lacked information about the patients’ current medications and smoking habits, which may modify the immunologic response. Most AAA patients would be on statins, antiplatelet medication, and/or antihypertensive drugs. Control thrombi were from samples from random healthy blood donors and not the included patients. This precluded a direct comparison of differences within the patients, who may have differences in circulating proteins as well. AAA development is a gradual process, and many patients have asymptomatic AAA before getting their diagnosis. We considered that thrombi from healthy controls would be more useful for comparison than thrombi from the patients’ own blood because the true duration of disease was unknown. The sex and age of the blood donors were not accessible, and differences in distribution from the AAA patients may have influenced the results. We cannot exclude that intraoperative factors may have affected the patients’ ILT, nor that misalignment of peptide sequences to proteins following mass spectrometry may have introduced errors. However, many previous studies have indicated activation of the investigated defense systems in the ILT, supporting that our findings were not spurious. Clot retraction in the control thrombi may not have been complete after 2 h. This was adjusted for by using exactly 200 ug protein from all samples as starting material for proteomic analysis. We did not have access to samples from the walls of the AAA, which would have strengthened the study. To verify the gradual steps of defense system changes in the ILT, an animal model would probably be necessary because longitudinal sampling is impossible in humans.

However, a strength of the study is the simultaneous investigation of a broad selection of relevant proteins including inhibitors and regulators, as well as a much more comprehensive analysis of antibodies than in previous studies. Proteomics permits such detection as well as detailed characterization of antibody diversity, whereas alternative approaches would have involved many different methods with results that were not necessarily comparable.

In conclusion, the present study supports an important role of complement and antibodies in the defense system activation in ILT from AAA. The biological processes taking place in the ILT may have implications for AAA development, growth, or rupture and may help explain that the ILT can have both detrimental and protective effects. Future research is warranted to clarify the influence of these mediators on the underlying aortic wall and their potential systemic effects, as well as the potential effects of complement and B cell inhibitors as treatments for AAA.

## Supplementary Information

Below is the link to the electronic supplementary material.

Supplementary Material 1

## Data Availability

The data generated or analyzed during this study are included in this published article, including in Supplementary Table S2 online.
