# Supplementary material for: Complement and diverse enrichment of antibodies in intraluminal thrombi from abdominal aortic aneurysms may contribute to increased inflammation
Source: Sci Rep. 2025 Nov 29;16:220. doi: 10.1038/s41598-025-29506-0 (PMC12770350; doi:10.1038/s41598-025-29506-0)
Supplement: Supplementary file 1 — Supplementary Material 1 [file 41598_2025_29506_MOESM1_ESM.pdf]

Complement and diverse enrichment of antibodies in intraluminal thrombi from abdominal aortic aneurysms may contribute to increased inflammation

Authors: Vibeke Videm, Animesh Sharma, and Torbjørn Dahl

Supplementary Table S1 gives an overview of the pre-specified proteins that were investigated in the study. Protein numbers and corresponding gene names are from the Uniprot database [1]. Several proteins have more than one Uniprot number and corresponding gene names, and only the most common are stated. Given main functions are those relevant to the study and the list is not exhaustive.

Supplementary Table S1: Pre-specified proteins for investigation in the study

Protein name	Uniprot number	Gene name(s)	Main functions	References
**Complement system**				
C1	P02745, P02746, P02747, P00736, P09871	*C1QA, C1QB, C1QC, C1R, C1S*	Subunits of Complement C1, part of classical pathway	[2]
C2	P06681	*C2*	Complement C2, part of classical and lectin pathways	[2]
C3	P01024	*C3*	Complement C3, part of all initial pathways	[2]
C4	P0C0L4, P0C0L5	*C4A, C4B*	Subunits of Complement C4, part of classical and lectin pathways	[2]
C5	P01031	*C5*	Complement C5, part of terminal pathway	[2]
C6	P13671	*C6*	Complement C6, part of terminal pathway	[2]
C7	P10643	*C7*	Complement C7, part of terminal pathway	[2]
C8	P07357, P07358, P07360	*C8A, C8B, C8G*	Subunits of Complement C8, part of terminal pathway	[2]
C9	P02748	*C9*	Complement C9, part of terminal pathway	[2]
B	P00751	*CFB*	Complement factor B, part of alternative pathway	[2, 3]
D	P00746	*CFD*	Complement factor D, part of alternative pathway	[2, 3]

Mannose-binding lectin	P11226	*MBL2*	Activator of complement lectin pathway	[2, 3]
MBL-associated serine protease 1	P48740,	*MASP1*	Activator of complement lectin pathway, potential role in coagulation cascade?	[2, 3]
MBL-associated serine protease 2	O00187	*MASP2*	Activator of complement lectin pathway, activates proenzyme of complement factor D	[2, 3, 4]
C1 inhibitor	P05155	*SERPING1*	Inhibitor of complement classical and lectin pathways, inhibitor of coagulation factors Xia, XIIa, inhibitor of kallikrein	[2, 3, 4]
C4-binding protein	P04003, P20851	*C4BPA, C4BPB*	Cofactor for C3b and C4b inactivation, binds to anticoagulation protein S	[2, 3, 4]
Factor H	P08603	*CFH*	Accelerates complement convertase decay, cofactor for Factor I	[2, 4]
Factor H-related protein 1	Q03591	*CFHR1*	Inhibits C5 cleavage and terminal pathway activation	[4, 5]
Factor I	P05156	*CFI*	Degrades complement C3b and C4b	[2, 3]
Carboxypeptidase B2	Q96IY4	*CPB2*	Inactivator of complement C3a, C4a, C5a	[6]
Carboxypeptidase N	P15169, P22792	*CPBN1, CPBN2*	Inactivator of complement C3a, C4a, C5a	[6]
Clusterin	P10909	*CLU*	Prevents assembly of terminal complement complex	[2, 3]
Vitronectin	P04004	*VTN*	Prevents assembly of terminal complement complex, stabilizes plasminogen activator inhibitor-1	[2, 3, 7]

Coagulation and fibrinolysis				
Kininogen	P01042	*KNG1*	Participates in initiation of contact activation	[4]
Plasma kallikrein	P03952	*KLKB1*	Cleaves high molecular weight kininogen, activates coagulation factor XII, cleaves complement factors B, C3, and C5	[4]
Tissue factor	P13726	*F3*	Binds to coagulation factor VIIa as part of the tissue factor pathway to initiate coagulation	[8]
Factor V	P12259	*F5*	Coagulation factor V, part of prothrombinase complex that amplifies coagulation	[8]
Factor VII	P08709	*F7*	Coagulation factor VII, part of extrinsic factor tenase complex that initiates extrinsic coagulation pathway	[8]
Factor VIII	P00451	*F8*	Coagulation factor VIII, part of intrinsic factor tenase complex that amplifies coagulation	[8]
Factor IX	P00741	*F9*	Coagulation factor IX, part of intrinsic factor tenase complex that amplifies coagulation	[8]
Factor X	P00742	*F10*	Coagulation factor X, part of prothrombinase complex that amplifies coagulation	[8]
Factor XI	P03951	*F11*	Coagulation factor XI, part of contact activation (intrinsic coagulation pathway)	[8]
Factor XII	P00748	*F12*	Coagulation factor XII, part of contact activation (intrinsic coagulation pathway)	[8]
Factor XIII	P00488, P05160	*F13A1, F13B*	Coagulation factor XIII, crosslinks fibrin strands	[8]

Prothrombin	P00734	*F2*	Inactive form of thrombin, which converts fibrinogen to fibrin	[8]
Fibrinogen	P02671, P02675, P02679	*FGA, FGB, FGG*	Inactive form of fibrin, which becomes the fibrin clot	[8]
Plasminogen	P00747	*PLG*	Inactive form of plasmin, which mediates fibrinolysis	[8]
Tissue-type plasminogen activator	P00750	*PLAT*	Performs proteolytic cleavage of plasminogen to plasmin	[8]
Urokinase-type plasminogen activator	P00749	*PLAU*	Performs proteolytic cleavage of plasminogen to plasmin	[8]
Platelet glycoprotein Ib	P07359 P13224	*GP1BA GP1BB*	Interacts with thrombin to enhance platelet aggregation	[8]
Platelet glycoprotein IIbIIIa	P08514, P05106	*ITGA2B, ITGB3*	Platelet receptor for fibrinogen and von Willebrand factor, enhances platelet aggregation	[8, 9]
Fibronectin	P02751	*FN1*	Stabilizes platelet aggregates, contributes to clot formation	[10, 11]
von Willebrand factor	P04275	*VWF*	Supports platelet adhesion and aggregation	[4]
Antithrombin III	P01008	*SERPINC1*	Neutralizes thrombin, factor IXa, factor Xa and other coagulation proteases	[4, 12]
Protein C	P04070	*PROC*	Anticoagulant effect on the prothrombinase complex of coagulation	[8, 12, 13]
Protein S	P07225	*PROS1*	Anticoagulant effect on initial and propagation phases of coagulation alone or in combination with other proteins	[8, 13]

Protein Z-dependent proteinase inhibitor	Q9UK55	*SERPINA10*	Contributes to inhibition of coagulation factors IX, X, and XI.	[14]
Plasmin activator inhibitor 1	P05121	*SERPINE1*	Inhibitor of tissue-type and urokinase-type plasminogen inhibitors	[7]
Alpha-2-antiplasmin	P08697	*SERPINF2*	Inhibitor of plasmin	[7]
**Neutrophil granulocytes**				
Cathepsin G	P08311	*CTSG*	Serin proteinase from neutrophil primary granules, microbicidal properties, degradation of extracellular matrix components, degradation of tissue inhibitors of matrix metalloproteinases	[15, 16, 17]
Elastase	P08246	*ELA2*	Serin proteinase from neutrophil primary granules, microbicidal properties, degradation of extracellular matrix components, inhibitor of all matrix metalloproteinases	[15, 16, 17]
Myeloperoxidase	P05164	*MPO*	Peroxidase from neutrophil primary granules, catalyzes formation of hypochlorous acid and other reactive oxygen species	[15, 16]
Proteinase 3	P24158	*PRTN3*	Serin proteinase from neutrophil primary granules, microbicidal properties, degradation of extracellular matrix components	[15, 16]
Lactoferrin	P02788	*LTF*	Anti-inflammatory and anti-microbial iron-binding protein from neutrophil secondary granules	[15, 16]

Neutrophil gelatinase-associated lipocalin	P80188	*NGAL*	Antimicrobial peptide from neutrophil secondary granules	[15, 16]
Lysosome-associated membrane protein 2	P13473	*LAMP2*	Marker for neutrophil tertiary granules, various intracellular functions including autophagy and endosome-phagosome fusion	[15, 18]
Matrix metalloproteinase 8	P22894	*MMP8*	Collagenase from neutrophil tertiary granules, degradation of extracellular matrix components	[15, 16]
Matrix metalloproteinase 9	P14780	*MMP9*	Gelatinase from neutrophil tertiary granules, degradation of extracellular matrix components	[15, 16]
Tissue inhibitor of metalloproteinases 2	P16035	*TIMP2*	Found in neutrophil tertiary granules and macrophages, inhibitor of all matrix metalloproteinases	[15, 19, 20]
Calprotectin (S100A8/S100A9)	P05109, P06702	*S100A8, S100A9*	Found in neutrophil cytosol, marker of neutrophil activation in inflammation, increases leukocyte chemotaxis and cytokine secretion, anti-inflammatory effects	[21, 22]
**Other proteinases and inhibitors**				
Matrix metalloproteinase 12	P39900	*MMP12*	From macrophages, elastase function, activation of chemokines	[19, 23]
Tissue inhibitor of metalloproteinase 1	P01033	*TIMP1*	From macrophages, inhibitor of most matrix metalloproteinases	[19, 20]
Tissue inhibitor of metalloproteinase 3	P35625	*TIMP3*	From macrophages, inhibitor of all matrix metalloproteinases	[19, 20]
Alpha-1-antitrypsin	P01009	*SERPINA1*	Produced in liver, inhibitor of elastase	[24]

Alpha-1-antichymotrypsin	P01011	*SERPINA3*	Produced in liver, inhibitor of cathepsin G	[24]
Leukocyte elastase inhibitor	P30740	*SERPINB1*	From macrophages, inhibitor of neutrophil elastase	[24]

References

1) https://www.uniprot.org/, release 2024_04, last accessed 20.07.2024

2) Merle, N.S., Church, S.E., Fremeaux-Bacchi, V. & Roumenina, L.T. Complement system part I - Molecular mechanisms of activation and regulation. *Front. Immunol*. **6**, 262; 10.3389/fimmu.2015.00262 (2015).

3) Ricklin, D., Hajishengallis, G., Yang, K & Lambris, J.D. Complement: a key system for immune surveillance and homeostasis. *Nat. Immunol.* **11**, 785-797 (2010).

4) Conway, E.M. Reincarnation of ancient links between coagulation and complement. *J. Thromb. Haemost.* **13**, S121-S132 (2015).

5) Skerka, C., Pradel, G., Halder, L.D., Zipfel, P.F., Zipfel, S.L.H. & Strauß, O. Factor H-related protein 1: a complement regulatory protein and guardian of necrotic-type surfaces. *Br. J. Pharmacol*. **178**, 2823-2831 (2021).

6) Morser, J. *et al.* Carboxypeptidase B2 and N play different roles in regulation of activated complements C3a and C5a in mice. *J. Thromb. Haemost*. **16**, 991-1002 (2018).

7) Mutch, N.J. & Medcalf, R.L. The fibrinolysis renaissance. *J. Thromb. Haemost*. **21**, 3304-3316 (2023).

8) Adams, R.L. & Bird, R.J. Review article: Coagulation cascade and therapeutics update: relevance to nephrology. Part 1: Overview of coagulation, thrombophilias and history of anticoagulants. *Nephrology (Carlton)*. **14**, 462-470 (2009).

9) Broos, K., Feys, H.B., De Meyer, S.F., Vanhoorelbeke, K. & Deckmyn, H. Platelets at work in primary hemostasis. *Blood Rev*. **25**, 155-167 (2011).

10) Cho J, Mosher DF. Role of fibronectin assembly in platelet thrombus formation. *J. Thromb Haemost.* 2006 Jul;4(7):1461-9. doi: 10.1111/j.1538-7836.2006.01943.x. PMID: 16839338.

11) Wang, Y. *et al.* Plasma fibronectin supports hemostasis and regulates thrombosis. *J. Clin. Invest.* **124**, 4281-4293 (2014).

12) Rezaie, A.R. & Giri, H. Anticoagulant and signaling functions of antithrombin. *J. Thromb. Haemost.* **18**, 3142-3153 (2020).

13) Gierula, M. & Ahnström, J. Anticoagulant protein S - new insights on interactions and functions. *J. Thromb. Haemost.* **18**, 2801-2811 (2020).

14) Corral, J., González-Conejero, R., Hernández-Espinosa, D. & Vicente, V. Protein Z/Z-dependent protease inhibitor (PZ/ZPI) anticoagulant system and thrombosis. *Br. J. Haematol.* **137**, 99-108 (2007).

15) Rørvig, S., Østergaard, O., Heegaard, N.H. & Borregaard, N. Proteome profiling of human neutrophil granule subsets, secretory vesicles, and cell membrane: correlation with transcriptome profiling of neutrophil precursors. *J. Leukoc. Biol*. **94**, 711-721 (2013).

16) Faurschou, M. & Borregaard, N. Neutrophil granules and secretory vesicles in inflammation. *Microbes Infect.* **5**, 1317-1327 (2003).

17) Okada, Y. *et al.* Inactivation of tissue inhibitor of metalloproteinases by neutrophil elastase and other serine proteinases. *FEBS Lett.* **229**, 157-160 (1988).

18) Beertsen, W. *et al.* Impaired phagosomal maturation in neutrophils leads to periodontitis in lysosomal-associated membrane protein-2 knockout mice. *J. Immunol.* **180**, 475-482 (2008).

19) Newby, A.C. Metalloproteinase production from macrophages - a perfect storm leading to atherosclerotic plaque rupture and myocardial infarction. *Exp. Physiol*. **101**, 1327-1337 (2016).

20) Brew, K. & Nagase, H. The tissue inhibitors of metalloproteinases (TIMPs): an ancient family with structural and functional diversity. *Biochim. Biophys. Acta.* **1803**, 55-71 (2010).

21) Vogl, T., Gharibyan, A.L. & Morozova-Roche, L.A. Pro-inflammatory S100A8 and S100A9 proteins: self-assembly into multifunctional native and amyloid complexes. *Int. J. Mol. Sci*. **13**, 2893-2917 (2012).

22) Wang, S., Song, R., Wang, Z., Jing, Z., Wang, S. & Ma, J. S100A8/A9 in inflammation. *Front. Immunol.* 9, 1298; 10.3389/fimmu.2018.01298 (2018).

23) Khokha, R., Murthy, A. & Weiss A. Metalloproteinases and their natural inhibitors in inflammation and immunity. *Nat. Rev. Immunol.* **13**, 649-665 (2013).

24) Law, R.H. *et al*. An overview of the serpin superfamily. *Genome Biol*. **7**, 216; 10.1186/gb-2006-7-5-216 (2006).

Supplementary table S2 - Data for main tables 1, 2, and 3.

Complement and diverse enrichment of antibodies in intraluminal thrombi from abdominal aortic aneurysms may contribute to increased inflammation.

Authors: Vibeke Videm, Animesh Sharma, Torbjørn Dahl

Abbreviations: C=Control, ILT=intraluminal thrombus, F=fluid from ILT

	Uniprot	Protein name	Gene name	C1	C2	C3	C4	C5	C6
	**Complement system**								
1	A0A8Q3SI63	Complement C1q A chain	C1QA						
2	A0A8Q3SI33	Complement C1q B chain	C1QB						
3	P02747	Complement C1q q subcomponent subunit C/C	C1QC	21.13204		20.93572		21.55798	21.18457
4	B4DPQ0	Complement C1r	C1R						
5	P09871	Complement C1s subcomponent	C1S					16.64851	15.61084
6	B4E1Z4	Complement C2	C2	22.38575	22.72632	22.11545	23.00152	23.80521	24.08406
7	A0A8Q3SI22	Complement C3 (partial sequence)	C3						
8	V9HWA9	Complement C3 (complete sequence)	HEL-S-62p	20.43282	19.88393	20.03224	19.65404	18.02579	18.80911
9	A0A0G2JPR0	Complement C4-A	C4A						
10	A0A140TA29	Complement C4-B	C4B						
11	P01031	Complement C5	C5				17.52457		16.44391
12	B2R6W1	complement component 7	C7						
13	A0A8Q3WKN4	Complement C8 alpha chain	C8A						
14	B7ZA94	Complement component C8 beta chain	C8B		16.4962				
15	P07360	Complement component C8 gamma chain	C8G						
16	A0A8Q3SI95	Complement C9	C9	18.9409	17.68007	16.97021			18.76984
17	A0A8V8TP45	MBL associated serine protease 2	MASP2						
18	P04003	C4b-binding protein alpha chain	C4BPA				10.003		
19	P20851	C4b-binding protein beta chain	C4BPB						
20	A8K5T0	complement factor H	CFH						
21	B1AKG0	Complement factor H related 1	CFHR1						
22	P05156	Complement factor I	CFI						
23	Q96IY4	Carboxypeptidase B2	CPB2						
24	P15169	Carboxypeptidase N catalytic chain	CPN1						
25	P10909-2	Clusterin	CLU	18.80641	19.75315	18.59397	19.33335	20.598	19.50955
26	P04004	Vitronectin	VTN	10.85425		16.21787	10.93331	11.3695	17.09815
	**Coagulation and fibrinolysis**								
27	B4DPP8	Kininogen-1	KNG1	16.06065					
28	P03952	Plasma kallikrein	KLKB1	18.79318		16.60073	18.48453		18.57438
29	P12259	Coagulation factor V	F5						
30	A0A8F0WPP1	Factor IX	F9						
31	Q5JVE7	Coagulation factor X	F10						
32	Q8IZZ5	Coagulation factor XII	F12						
33	P00488	Coagulation factor XIII A chain	F13A1	10.26537					
34	P05160	Coagulation factor XIII B chain	F13B					17.1961	
35	P00734	Prothrombin	F2		17.05272	15.73361	17.08773		15.53488
36	P02671	Fibrinogen alpha chain	FGA	22.13725	22.71384	23.33306	22.87459	22.37496	21.70968
37	P02679-2	Fibrinogen gamma chain	FGG	22.32539	22.41256	23.35428	22.63679	22.26828	21.33426
38	A6PVI2	Plasminogen	PLG					17.23307	
39	E0D851	Platelet glycoprotein Ib alpha	GP1BA			17.77908			17.21848
40	P13224	Platelet glycoprotein Ib beta chain	GP1BB						
41	Q68CX6	Fibronectin (partial sequence)	FN1						
42	B7ZLE5	Fibronectin (complete sequence)	FN1	17.2034	14.76746	16.25136	16.58715	17.85543	16.69302
43	L8E853	von Willebrand factor	VWF		15.33408	15.37726	15.69396		15.00188
44	ANT3_HUMAN	Antithrombin-III	SERPINC1	19.76604	19.31266	20.13127	19.74476	20.82958	20.04916
45	Q8J009	Protein C	PROC						
46	G3V2W1	Protein Z-dependent protease inhibitor	SERPINA10						
47	P05121	Plasminogen activator inhibitor 1	SERPINE1						
48	P08697	Alpha-2-antiplasmin	SERPINF2						
	**Neutrophil granulocytes, other proteinases and inhibitors**								
49	CATG_HUMAN	Cathepsin G	CTSG	16.64002	14.91824	15.44182			
50	P05164	Myeloperoxidase	MPO	16.34633		15.85452		16.64271	
51	A0A161I202	Lactotransferrin	LTF			15.84541			15.77293
52	P13473	Lysosome-associated membrane glycoprotein 2	LAMP2						
53	P14780	Matrix metalloproteinase-9	MMP9						
54	P05109	Protein S100-A8	S100A8	19.19934	18.13809	19.13008	17.59546	18.29262	16.77767
55	P06702	Protein S100-A9	S100A9	18.75411	18.37384	18.86622	18.10838		17.20724
56	P39900	Macrophage metalloelastase	MMP12						
57	P01033	Metalloproteinase inhibitor	TIMP1						
58	P35625	Metalloproteinase inhibitor 3	TIMP3						
59	A0A024R6N5	Alpha-1-antitrypsin	SERPINA1	16.53487				18.33866	
60	P01011	Alpha-1-antichymotrypsin	SERPINA3						
61	P30740	Leukocyte elastase inhibitor	SERPINB1			16.1649			

Protein abundance, Log 2 normalized values

	C7	ILT1	ILT2	ILT3	ILT4	ILT5	ILT6	ILT7	F1	F2	F3	F4	F5	F6
1		18.89603	20.17546	20.15016	20.53109	18.70141	20.31426		21.0747	21.05507		19.59417	20.36373	19.71418
2		18.23736	19.46774	19.56013	19.82976	18.75154	19.51304	19.7652	20.4522	20.37062		19.44044	19.771	19.59726
3		20.84767	22.2969	22.45133	22.08914	20.94175	21.84332	22.30029	22.86691	22.75158	17.53901	21.82845	21.9212	21.45384
4		19.37262	21.51844	20.07487	21.79736	20.68006	21.74113	18.06077	22.26947	21.99071	16.45896	21.05623	21.72062	21.32831
5	17.16411	17.56434	19.60261	16.20826	20.38439	18.84041	19.28158	17.91795	21.02963	20.72407	17.52504	20.0515	20.60575	20.23151
6	24.29614	21.03706	22.72613	22.14679	21.56989	22.29379	21.91252	22.36786	22.44932	22.96231	23.73007	22.2313	22.45497	22.87228
7			18.23478		17.60334	17.85025	16.98637	17.93397	18.77187	19.10303		19.19635	19.24254	19.12337
8	19.61999	25.73517	26.5589	25.28967	26.42501	26.55594	26.72924	25.23928	26.69224	26.91644	23.84881	26.53668	26.66318	26.81356
9			17.31986			18.40165	16.48317		17.62088	19.80513		17.57507	19.68925	18.85128
10		16.45886		16.46583			16.30754		17.45042	17.07566			16.4547	17.61281
11	16.86862	22.59579	23.7761	22.95941	23.40881	22.63221	22.58093	22.05663	23.91848	24.09556	18.36616	22.84008	22.95262	22.76096
12		18.52555	20.72492	20.71074	20.79192	20.0297	19.94496	19.18072	21.1677	21.34147		19.93447	20.52779	19.78974
13			17.72868	15.77256	18.89351	18.01048			18.66979	19.46939				
14		19.66246	21.25981	21.05117	20.99479	20.70231	20.63773	18.69167	21.20158	21.50692	17.43448	19.84183	20.6302	19.5108
15		18.29519	20.72443	20.7868	20.51252	20.31345	19.86252	19.52533	20.91203	21.27062		20.05558	20.57081	19.5146
16	16.86072	22.3484	23.79516	25.23927	23.82806	23.43144	23.23744	23.33527	23.93445	24.09133	19.68633	22.45581	22.96255	21.66318
17			17.05629	15.58701	18.04437	17.22139	18.74266	16.56574	17.16143	18.07853		16.7241	17.33717	17.44396
18		22.58567	23.78291	23.05144	23.64052	22.59193	22.6276	21.45263	25.13414	25.18043	16.82813	23.74607	24.25909	23.80527
19		17.40366	18.90697	18.87333	18.91636	15.23754	17.50947	17.80891	20.77137	20.4159		19.27168	19.72974	19.03049
20		20.87137	21.74486	20.54429	21.38412	20.96687	20.31416	17.63726	22.11894	22.15875	16.65508	21.49164	21.82373	21.23773
21			18.91518	18.47936	19.10418	18.8663	18.48259						17.22692	17.94115
22		19.08678	20.74687	19.78837	20.06452	20.06109	20.57608	17.92432	20.94635	21.37743		20.24243	20.90036	21.02409
23		20.30316	21.47581	21.10119	21.19471	21.37307	20.90585	20.4001	18.88427	19.35446	14.99432		17.83671	18.04207
24		17.0954	17.11801		16.32305	15.57785	17.44435	16.72218	18.54144	18.57644		17.16264	18.47639	17.98549
25	19.31566	24.92125	24.93914	24.09629	24.65892	24.20391	24.24007	25.19634	23.53205	23.97521	21.58827	22.23621	22.90071	22.44224
26		24.46097	25.79023	25.52167	25.55801	24.70531	25.20106	23.8158	23.82841	24.50625	18.85513	23.14017	23.12332	22.97408
27	15.57734	22.1176	23.64254	22.21901	22.44825	22.71297	22.1344	21.44597	23.31871	24.11076	20.90619	23.20299	23.4852	23.46609
28	18.32056	19.40059	20.86735	20.82895	20.19848	19.43137	20.07755	18.36536	21.00397	21.31361	18.16754	20.7297	20.78307	20.65348
29	16.31757	18.55271	19.56858		19.43834	19.51288	19.83516	17.41433	18.64824	18.9687	18.23753	17.63873	18.93211	17.15949
30		19.4039	20.85341	21.60532	19.85112	18.73931	19.6174	20.29289	19.20445	19.59881		16.21123	17.78367	17.56104
31		18.56856	19.9195	19.61867	18.98345	17.36447	19.61688	19.18837	18.39252	18.61843		17.48743	17.80171	17.15224
32			18.62574		15.5666	16.98455	18.16664	17.61437	18.98749	19.24416		18.35426	18.53005	18.78331
33		20.10094	19.80157	18.7189	19.72433	20.4534	20.41896	20.52197	16.96204	17.96639	16.14063	12.98561	16.75935	
34		18.12751	19.28953	17.42433	19.10537	19.02887	19.27987		17.70612	18.78259		17.26315	18.25271	17.67211
35	15.29534	24.25377	24.83619	22.96152	24.68411	24.49864	24.73415	22.99939	23.82117	24.41667	19.07201	23.25557	23.38569	23.3094
36	22.36275	28.87821	28.9898	26.67079	28.85653	29.20994	28.1359	28.25207	26.97685	27.12646	23.05301	26.37625	26.62419	25.82941
37	21.84917	29.39156	29.56294	27.18883	29.68448	29.76617	28.83617	28.50254	27.41673	27.78502	23.02458	26.93011	27.35721	26.29598
38		24.31352	25.4655	24.05065	25.65856	25.46857	25.48689	22.66183	23.59298	24.45229	15.86981	22.42401	23.57785	22.88431
39		18.73686	11.48585	17.52472		10.3031	17.07547	17.01217	19.75265	17.03469	19.0337	18.84279	17.61516	17.67891
40		19.14982		15.38756		19.16912	19.32244	17.34845	15.36094			15.17588		
41		17.95184	17.9136	18.96005		17.01755	15.16158	19.87353	17.33131			16.9788	17.70464	17.14388
42	16.7841	23.87042	24.36419	23.72652	23.5621	24.06983	23.26728	24.25951	24.46292	25.02525	20.06969	24.1267	24.42071	24.71106
43	16.34512	16.84651	16.53028		16.01488	15.94144	17.16186	18.90248	20.40177	20.58799		19.89733	20.61847	19.26782
44	20.16777	21.5951	21.88724	21.70456	21.28041	21.75788	21.53952	21.58017	22.58332	22.82368	20.70894	22.32332	22.31108	22.70255
45			16.02819				15.6478		15.87097	16.3549		16.15231		16.07857
46			18.49566	18.96392	17.45786	17.25598			15.40562	16.57751				
47			14.62675	15.46135	16.05333	14.95877	16.59912		17.40858	16.79535		15.53352		16.82164
48		22.82684	23.09937	21.10053	22.88479	22.85554	22.55684	21.32057	21.93897	22.26538	15.06619	21.62458	21.74704	21.74495
49	15.70521	20.51603	17.76354	20.35317	20.21982	19.32492	19.57351	21.77033	18.30038	17.35127		16.85047	16.10179	
50	16.96204	20.28933	18.97184	21.19883	18.7509		19.05401	21.08564	21.53894	18.85688	17.0736	19.60551	17.66153	17.43954
51		20.94422	20.45069	21.60248	20.13776	18.7138	18.85605	20.89575	20.8397	19.61585	18.75676	20.04543	19.78736	20.23497
52		16.26335	15.98181	16.84117				18.40991	16.11914	15.54024			15.91564	15.80415
53			16.47357	16.33934	15.40832			16.98417		18.28954				
54	17.5704	22.37589	20.6477	22.27394	20.64974	19.07743	21.46552	22.2918	24.54495	22.40526	19.18391	23.14294	21.9569	21.64175
55	18.50796	21.47575	19.40268	21.51224	18.83018	17.14842	19.99966	22.45639	23.7804	21.26255	18.54007	22.01665	20.47458	20.52193
56		17.96338	18.86109	21.13031	17.0941		13.84065	20.6304	18.84449	18.81185		15.50941		16.75596
57		19.6107	19.31385	19.38382	19.70251	17.72494	18.92372	18.01755	20.50295	19.53181		18.5745	18.89375	18.48218
58		18.74781	16.01488	21.58534	15.60867			19.28572	17.04585	16.01561		15.69212		14.76119
59					20.38717	19.87896	17.86635	18.96817	18.05296	19.96575		20.4841	20.44863	20.94079
60		22.91868	23.69355	23.14044	22.92922	23.86787	22.66574	23.45126	24.24865	24.34162	21.99077	23.71595	24.17395	24.02685
61		17.59163	14.94839	17.55793	15.34753	14.07793		18.88828	15.01735					

	F7	Protein FDR confidence	Posterior Error Probabilty Score	Coverage [%]	Peptides (number)	Isoforms (number)	Peptide spectrum matches (number)	Unique Peptides (number)	Amino acids (number)
1		High	5.079	28	2	2	11	2	120
2		High	18.561	11	2	2	26	2	248
3	20.9459	High	60.292	35	6	12	69	6	245
4		High	80.515	27	13	17	108	12	719
5		High	57.851	22	10	12	43	10	688
6	22.88152	High	95.094	17	19	21	176	19	1266
7	17.53444	High	112.173	70	9	12	171	1	198
8	24.08674	High	627.32	55	74	101	989	66	1663
9		High	353.213	40	53	57	544	2	1744
10		High	351.82	43	53	58	542	1	1698
11	19.30656	High	164.077	25	35	39	264	31	1676
12		High	73.644	22	12	14	92	12	843
13		High	54.91	19	6	9	32	6	567
14	16.97505	High	38.892	19	9	9	49	9	539
15		High	42.869	40	6	6	52	6	202
16	19.60004	High	75.472	24	13	18	144	13	535
17		High	4.28	3	2	2	7	2	651
18	16.23837	High	161.962	45	18	26	256	18	597
19		High	22.624	21	4	5	32	4	252
20	16.78544	High	353.749	47	40	63	505	2	1231
21		High	44.713	38	8	12	52	1	271
22		High	23.989	11	6	6	36	6	583
23		High	25.598	18	6	6	28	6	423
24		High	9.331	8	3	3	7	3	458
25	20.45925	High	103.378	29	15	23	246	15	501
26	17.80739	High	92.563	37	13	16	321	11	478
27	20.05692	High	87.036	36	13	17	133	13	415
28	18.40946	High	33.516	16	9	9	46	9	638
29		High	22.993	3	6	7	21	6	2224
30		High	13.48	9	4	4	25	4	461
31		High	9.854	6	3	3	4	3	488
32		High	29.934	10	4	5	13	4	615
33		High	41.625	16	10	10	38	9	732
34	16.09024	High	31.931	13	6	6	25	6	661
35	20.08666	High	165.854	40	19	25	238	19	622
36	22.81636	High	303.538	33	27	58	1011	27	866
37	22.26535	High	569.234	82	35	89	2196	23	437
38	17.16344	High	351.399	55	35	60	442	35	827
39	17.57399	High	18.696	8	5	5	13	5	665
40		High	6.536	10	2	2	6	2	206
41		High	238.803	39	27	38	340	2	2146
42	19.73871	High	451.582	34	54	78	673	29	2240
43	16.45348	High	52.547	5	11	12	33	11	2715
44	20.69421	High	73.013	28	11	12	130	11	464
45		High	2.717	4	1	1	3	1	211
46		High	9.605	5	2	2	8	2	484
47		High	5.859	4	2	2	6	2	402
48	14.37683	High	66.551	23	8	11	107	8	491
49	19.54197	High	7.665	10	3	3	21	3	255
50	18.02624	High	64.161	21	13	14	59	12	745
51	19.95506	High	74.311	26	14	17	44	14	711
52		High	1.692	2	1	1	4	1	410
53		High	18.692	9	5	5	8	5	707
54	20.20051	High	21.658	40	4	5	50	4	93
55	20.14674	High	37.259	51	5	7	51	5	114
56		High	13.783	11	6	6	17	6	470
57		High	27.414	32	4	6	19	4	207
58		High	19.53	23	4	4	8	4	211
59		High	259.515	58	25	43	581	2	418
60	22.09462	High	45.19	25	10	11	129	10	423
61		High	8.414	9	3	3	4	3	379

												Pr
	MW [kDa]	Score Sequest HT	C1	C2	C3	C4	C5	C6	C7	ILT1	ILT2	ILT3
1	13.4	31.21								76.1	184.6	181.4
2	26.2	90.84								64.8	152.1	162.2
3	25.8	214.25	73.7		64.4		99.1	76.5		60.6	165.3	184
4	81.8	357.65								45.3	200.4	73.7
5	76.6	139.88					17.9	8.7	25.6	33.7	138.6	13.2
6	140.9	515.8	70.3	89	58.3	107.8	188.1	228.2	264.4	27.6	89	59.6
7	21.8	645.14									157.4	
8	187	3415.58	2.6	1.8	2	1.5	0.5	0.9	1.5	103.7	183.6	76.2
9	192.8	1780.97									106.3	
10	187.5	1792.05								217.6		218.7
11	188.2	753.34				3.4		1.6	2.2	115.7	262.3	148.9
12	93.5	324.56								47.3	217.3	215.2
13	63.1	133.2									210.5	54.2
14	61.3	137.45		9.5						85.2	257.7	223
15	22.3	167.67								41.5	223.6	233.5
16	60.6	452.49	6.6	2.7	1.7			5.9	1.6	69.9	190.6	518.6
17	71.7	14.6									141.7	51.2
18	67	836.09				0				72.6	166.5	100.3
19	28.3	90.03								49.7	141	137.7
20	138.9	1765.12								126.5	231.8	100.8
21	30.8	189.93									388.4	287.2
22	65.7	107.37								67.6	213.6	109.9
23	48.4	80.83								163.5	368.6	284.3
24	52.3	19.23								131.1	133.2	
25	57.8	793.06	4	7.6	3.4	5.7	13.7	6.4	5.6	274.5	278	155
26	54.3	1021.64	0		0.5	0	0	0.9		148.9	374.2	310.6
27	46.5	431.31	1.3						1	89	256.2	95.5
28	71.3	141.35	47.7		10.4	38.5		41	34.4	72.7	200.8	195.5
29	251.5	61.44							29.1	137	276.9	
30	51.9	83.19								130.7	357	601.2
31	54.7	10.53								146.4	373.3	303.1
32	67.7	37.57									251.1	
33	83.2	93.38	0.3							286.1	232.5	109.8
34	75.5	76.71					71.6			136.6	305.7	83.9
35	70	810.54		1.3	0.5	1.4		0.5	0.4	194.7	291.6	79.5
36	94.9	3262.91	2.8	4.2	6.4	4.7	3.3	2.1	3.3	300.9	325.1	65.1
37	49.5	7354.43	2.2	2.3	4.4	2.7	2.1	1.1	1.5	288.2	324.6	62.6
38	92.4	1364.48					1.1			146.2	324.9	121.9
39	72.8	34.08			120.9			82		234.8	1.5	101.3
40	21.7	14.13								577.4		42.6
41	235.3	1169.1								190.6	185.7	383.5
42	246.5	2244.94	1.3	0.2	0.7	0.9	2.1	0.9	1	134.6	189.5	121.8
43	298.2	100.48		11.6	11.9	14.8		9.2	23.3	33	26.5	
44	52.6	374.23	28.9	21.1	37.2	28.5	60.4	35.1	38.2	102.6	125.6	110.7
45	23.3	6.99									347.4	
46	55.1	28.4									571.3	790.3
47	45	14.63									73.7	131.5
48	54.5	333.4								251.3	303.5	75.9
49	28.8	55.6	19.6	5.9	8.5				10.2	287.1	42.6	256.4
50	83.8	174.25	13.6		9.6		16.7		20.8	208.7	83.7	392
51	78.3	139.91			6.8			6.5		233.8	166.1	369
52	44.9	10								195	160.5	291.1
53	78.4	22.81									286.3	260.9
54	10.8	127.28	17.3	8.3	16.5	5.7	9.2	3.2	5.6	156.3	47.2	145.7
55	13.2	155.19	22.2	17.1	24	14.2		7.6	18.7	146.7	34.9	150.4
56	54	40.1								91.1	169.7	818
57	23.2	64.91								225.5	183.5	192.7
58	24.1	23.82								199.9	30.1	1428.9
59	46.7	1946.16	20.5				71.6					
60	47.6	439.8								96.9	165.8	113
61	42.7	11.43			143.8					386.6	61.9	377.7

rotein abundance, scaled

	ILT4	ILT5	ILT6	ILT7	F1	F2	F3	F4	F5	F6	F7	C1	C2	C3
1	236.2	66.5	203.3		344.3	339.7		123.4	210.4	134.1				
2	195.5	92.6	157	187	301	284.4		149.3	187.7	166.4				
3	143.2	64.6	120.7	165.7	245.5	226.6	6.1	119.5	127.4	92.2	64.8	1035253		817043.4
4	243.2	112.1	233.9	18.2	337.3	278.1	6	145.5	230.6	175.7				
5	238.3	81.7	110.9	43.1	372.7	301.5	32.8	189.2	277.8	214.3				
6	39.9	66	50.7	69.5	73.5	104.9	178.6	63.2	73.8	98.5	99.2	2468601	3257798	1850885
7	101.6	120.5	66.2	127.7	228.3	287.2		306.4	316.4	291.3	96.8			
8	167.3	183.2	206.6	73.6	201.4	235.2	28.1	180.8	197.4	219	33.1	637613.7	454232.2	436786.4
9		225	59.5		130.9	595.1		126.8	549.2	307.2				
10			196		432.7	333.7			217	484.3				
11	203.3	118.7	114.5	79.6	289.5	327.3	6.2	137.1	148.2	129.8	11.8			
12	227.6	134.2	126.5	74.5	295.4	333.2		125.6	189.5	113.6				
13	471.9	255.9			404.1	703.4								
14	214.4	175.1	167.4	43.4	247.5	305.8	18.2	96.4	166.5	76.7	13.2		43398	
15	193.1	168.2	123	97.4	254.7	326.5		140.7	201	96.7				
16	195	148.1	129.5	138.6	209.9	234	11	75.3	107	43.5	10.4	226696.9	98594.3	52300.45
17	281.1	158.9	456	100.9	152.4	287.8		112.6	172.2	185.4				
18	150.9	72.9	74.8	33.1	424.8	438.7	1.3	162.3	231.6	169.1	0.9			
19	141.9	11.1	53.5	65.9	513.4	401.3		181.5	249.4	153.6				
20	180.5	135.2	86	13.4	300.4	308.8	6.8	194.5	244.8	163.1	7.4			
21	442.8	375.5	287.8						120.5	197.7				
22	133.1	132.8	189.8	30.2	245.3	330.7		150.6	237.6	258.9				
23	303.4	343.3	248.3	174.9	61.2	84.7	4.1		29.6	34.1				
24	76.8	45.8	167	101.2	357.2	366		137.4	341.5	243				
25	228.9	167	171.2	332.2	104.8	142.5	27.2	42.7	67.7	49.2	12.5	206519.4	414868.2	161178.7
26	318.6	176.4	248.7	95.2	96.1	153.7	3.1	59.6	58.9	53.1	1.5	833.9131		31047.71
27	112	134.5	90.1	55.9	204.7	354.5	38.5	188.9	229.8	226.7	21.3	30789.69		
28	126.3	74.2	116.2	35.4	220.8	273.6	30.9	182.5	189.4	173.1	36.5	204633.9		40483.87
29	253	266.5	333.2	62.2	146.3	182.7	110.1	72.7	178.2	52.1				
30	178.2	82.5	151.6	242.1	113.8	149.6		14.3	42.5	36.4				
31	195.1	63.5	302.7	224.9	129.5	151.5		69.2	86	54.8				
32	30.1	80.5	182.6	124.6	322.6	385.5		208	235	280.1				
33	220.3	365.2	356.6	383	32.5	65.1	18.4	2.1	28.2			554.4367		
34	269.1	255.2	303.7		102	215.1		75	149	99.6	33.3			
35	262.4	230.7	271.6	81.6	144.3	218	5.4	97.5	106.7	101.2	10.8		63826.47	22194.93
36	296.4	378.7	179.9	194.9	80.5	89.3	5.3	53.1	63.1	36.4	4.5	2077985	3229741	4304441
37	353.1	373.7	196.1	155.6	73.3	94.6	3.5	52.3	70.4	33.7	2.1	2367435	2621043	4368202
38	371.4	325.6	329.7	46.5	88.7	161	0.4	39.5	87.8	54.3	1			
39		0.7	74.2	71	474.7	72.2	288.4	252.7	107.9	112.8	104.9			91622.21
40		585.2	650.8	165.7	41.8			36.7						
41		99.8	27.6	722.3	124			97.1	160.6	108.9				
42	108.7	154.5	88.6	176.3	202.9	299.7	9.7	160.8	197.1	241	7.7	67983.88	13093.92	31776.89
43	18.5	17.6	41.1	137.2	387.9	441.3		273.4	450.7	176.7	25.1		19392.75	17337.28
44	82.5	114.9	98.7	101.6	203.6	240.5	55.5	170	168.5	221.1	55	401642.4	305709.7	467820.3
45			266.9		311.6	435.7		378.6		359.8				
46	278.2	241.9			67.1	151.2								
47	198.2	92.8	289.3		507.1	331.5		138.2		337.6				
48	261.5	256.3	208.4	88.4	135.8	170.3	1.2	109.2	118.9	118.7	0.7			
49	233.8	125.7	149.4	684.8	61.8	32		22.6	13.5		146.1	46005.73	14536.47	18130.76
50	71.8		88.6	362.4	496.2	77.3	22.5	129.9	33.8	28.9	43.5	37532.22		24135.09
51	133.7	49.8	55	226.1	217.5	93.1	51.3	125.4	104.9	143	117.8			23983.18
52				863.6	176.5	118.2			153.3	141.9				
53	136.8			407.9		1008.1								
54	47.3	15.9	83.2	147.5	703.1	159.5	17.1	266	116.9	94	34.6	271172.6	135434.6	233717.4
55	23.4	7.3	52.7	289.5	724.7	126.5	19.2	213.4	73.3	75.7	58.4	199167.4	159476.2	194653.1
56	49.9		5.2	578.4	167.7	164		16.6		39.4				
57	240.3	61	140.1	74.7	418.5	213.5		109.9	137.2	103.1				
58	22.7			290.2	61.4	30.1		24		12.6				
59	296.3	208.3	51.6	110.8	58.8	221.2		316.9	309.2	434.9		42772.02		
60	97.6	187.1	81.3	140.2	243.7	259.9	50.9	168.4	231.4	208.9	54.7			
61	81.6	33.8		949.7	64.9									29928.55

Protein abundance

	C4	C5	C6	C7	ILT1	ILT2	ILT3	ILT4	ILT5	ILT6	ILT7	F1	F2	F3
1					157862.2	720184.7	747700	1049539	358686.5	824753.4		1981292	2178757	
2					99999.8	440960.6	496717	645469.3	371369.5	473298.7	343690.9	1286934	1355717	
3		1144189	939363.8		610632.9	3133745	3685086	3090417	1694816	2380226	1992073	6862090	7061666	59127.09
4					219658	1826929	709677.3	2524539	1413671	2217455	105458.3	4535355	4167383	27967.78
5		38071.46	19723.03	51504.39	62719.14	484173.4	48651.64	948064.7	394964.4	403141.4	95518.75	1920349	1732069	58557.34
6	3478230	5432254	7009138	7224393	696292.3	4219632	2983817	2156288	4326420	2497188	2087590	5137469	8172320	4319964
7						187604.5		137929.9	198833.8	82135.4	96585.65	401539.5	563100	
8	341717.3	98900.99	181029.3	282579.1	18074422	60124534	26355651	62408568	83016084	70376159	15276644	97273569	1.27E+08	4690556
9						99500.45			291392.3	57949.78		180819.4	916089.3	
10					29148.52		58161.3			51307.68		160668.2	138129.2	
11	78096.9		35136.03	41965.63	2051246	8736695	5240784	7713978	5470197	3968827	1682498	14223618	17926077	104902
12					122110.4	1054021	1102754	1257526	900677.7	638490.8	229203.9	2113224	2657189	
13						132096.5	35969.81	337317.3	222190.3			374107.9	725890.1	
14					268532.9	1527103	1396229	1447387	1435644	1032050	163306.3	2163437	2980090	54994.45
15					104089.7	1053663	1162454	1036111	1096444	603030.8	291044	1770030	2529847	
16			176168.3	41736.37	1728005	8852859	25450852	10315336	9519081	6255908	4081920	14381951	17873653	261934.1
17						82886.22	31628.75	187249	128583.3	277476.5	37413.98	131503.3	276809.4	
18	425.0272				2036903	8778017	5585978	9057954	5319592	4099301	1106962	33033955	38024670	36123.39
19					56108.61	298945.4	308575.4	342702.2	32507.65	118031.9	88565.78	1605601	1398939	
20					620745.7	2137386	982590.1	1895767	1724590	824697.7	78630.69	4085985	4682177	32040.35
21						300651.8	234836.2	390351.9	402114.8	231707.1				
22					180176.9	1070182	581858.1	759532	920491.5	988876.3	95941.63	1812632	2724256	
23					418665.5	1773737	1445491	1662518	2285414	1242831	533693.7	434073.1	670304.3	10133.43
24					45314.38	86508.98		56787.33	41155.69	112822.8	41699.21	342264.2	390899.4	
25	273608.3	588186	294171.8	228837	10281346	19563896	11524680	18348422	16260438	12534634	14828719	10881361	16491346	978887.7
26	809.9651	980.522	55296.53		7472929	35290622	30953647	34217875	23018106	24400590	5695315	13362766	23829619	147223.3
27				17146.71	1472540	7964152	3136982	3963876	5785138	2912346	1101867	9385581	18116002	610108
28	151918.4		153845.9	114807.7	223957.7	1163390	1196916	833433.5	594915.6	699952	130248.2	1886497	2606363	91409.55
29				28642.56	124431	472884.1		492094.5	629494.7	591700.6	67372.59	368561.2	513034.3	95953.6
30					224471.7	1152205	2050096	655099.5	368232.8	508804	495471.3	541934.5	794018.9	
31					125805.7	603107.2	517287	359013.7	141989	508619.9	230421.9	308697	402444	
32						245999.1		33615.29	109116	186135.1	77393.24	466266.7	620969.2	
33					363907.7	555766.2	277252.3	599985.1	1208132	886834.2	580735	114528.8	256108	22430.05
34		55646.8			92668	389720	113023.6	390674.4	450080.5	402662.9		191824.3	450945.3	
35	57694.15		18711.46	14102.27	6473185	18216440	5248437	18671627	19945904	17654111	3234114	13295946	22395001	171105.8
36	3185298	2015735	1351784	1891441	1.6E+08	3.24E+08	68648620	3.37E+08	5.23E+08	1.87E+08	1.23E+08	1.18E+08	1.47E+08	2701873
37	2701247	1872053	1042063	1324918	2.28E+08	4.82E+08	98305270	5.98E+08	7.68E+08	3.03E+08	1.47E+08	1.61E+08	2.31E+08	2649150
38		57091.33			6746931	28177654	11165819	36687781	39068922	29746940	2559404	11350810	22954680	18591.14
39			60106.36		141371.6	1744.264	121168.8		1063.093	87368.18	50982.56	792451.5	134262.3	166621.5
40					188224.5		27544.93		496032.8	414723	64365.57	37751.64		
41					82044.14	150161.1	327692.5		111640.5	23185.33	370490.5	147936.7		
42	40779.61	87885.92	41758.16	39577.63	4962717	13133469	8919040	8578743	14817334	6386639	7746228	20744575	34146870	341660.7
43	21956.61		12931.77	29194.78	38133.94	57562.27		45865.1	52951.64	92759.36	188999.7	1242730	1576172	
44	363894.9	690598.2	427603.4	413083.7	1025130	2359073	2196084	1764267	2984025	1928257	1209283	5637530	7423538	532145
45						40643.6				32477.47		53761.31	83813.84	
46						224789.5	328572.7	124699.1	131703.5			38939.02	97797.45	
47						15385.74	28990.29	47104.14	26795.89	62799.72		156075.8	113738	
48					2407523	5465512	1444827	5364539	6386061	3903087	1010136	3606784	5041346	10651.04
49				18735.79	485231	135327.6	860676.7	845853.5	552597.9	493558.1	1379657	289597.8	167206.2	
50		37918.6		44772.88	414671.5	312694.3	1546709	305567.5		344313.4	858336.9	2733377	474772.8	42823.75
51			22068.13		652898.7	871559	2046058	799084.1	361780.3	300164.4	752483.6	1683475	803451	137519.6
52					25454.35	39357.68	75443.66				134333.4	63852.45	47651.54	
53						55343.32	53278.8	30122.39			50002.66		320405.2	
54	82030.11	118994	44281.47	68257.84	1761246	999085.8	3258735	1139503	465485.6	1831844	1980382	21958311	5554641	184905.4
55	117051.7		59639.95	130732.7	943735.9	421516.2	1922005	322829.8	122241	663163.5	2219714	12925448	2515729	118341.1
56					82703.48	289587	1474959	96908.29		9280.581	626062.7	422267.3	460182.5	
57					259069.2	396346.5	439578.4	590978.1	182291.4	314579.9	102346	1333016	757985.4	
58					142449.5	40270.48	2021901	34610.02			246507.1	121379.1	66248.91	
59		122852.7						949893.5	811318.7	151157.9	197804.2	243957.3	1023977	
60					2565768	8250815	5941405	5532306	12881708	4209116	4423643	17881408	21259658	1293898
61					63916.66	19228.33	123989.8	28879.55	14551.5		187148.7	29751.18		

	F4	F5	F6	F7	C1	C2	C3	C4	C5	C6	C7	ILT1	ILT2	ILT3
														Prot
1	407139.8	1002312	549292.3									487833.4	1184179	1163599
2	365988.2	664618.1	506531.6									309024.2	725058.6	773010.1
3	1915711	2950171	1834398	777435.5	2298155		2005768		3087446	2383375		1887007	5152726	5734872
4	1121681	2567243	1681537									678797.9	3003967	1104427
5	559004.5	1185379	786207.2						102731	50041.71	146862.4	193817.7	796112.2	75713.55
6	2532800	4270976	4903271	2974026	5480038	6939117	4543757	8397422	14658239	17783745	20600025	2151716	6938218	4643531
7	309020.8	460776.7	364713.2	73065.84									308472.6	
8	50078003	78944938	75323362	6857302	1415436	967515.7	1072272	825001.2	266871.6	459311.8	805761.4	55854456	98861021	41015672
9	100446.4	628003.1	302026.7										163605.7	
10		66721.52	128005.6									90076.16		90512.84
11	3862431	6030251	4539155	249560.3				188547.8		89147.94	119663	6338859	14365493	8155909
12	515446.9	1123021	578827.6									377351.4	1733097	1716147
13													217202.5	55977.59
14	483389.9	1205638	477068.7	49581.47		92437.84						829833.4	2510971	2172866
15	560585.1	1157017	478327.1									321663.1	1732507	1809055
16	2959265	6071882	2120850	305858.1	503243.6	210006.1	128392.9			446978.1	119009.3	5339965	14556498	39607590
17	55688.77	123003	113867.4										136287.4	49221.88
18	7237534	14914921	9361465	29754.89				1026.135				6294538	14433439	8693113
19	325586.2	645877.5	341974.2									173389.5	491547.2	480216.8
20	1516838	2757436	1579207	43475.21								1918258	3514441	1529144
21		113953.5	160719.6										494353	365461.1
22	638101.4	1453930	1361839									556791.5	1759670	905509.8
23		173897.3	172364.5									1293780	2916504	2249528
24	75471.04	270929.2	165735.5									140032.7	142244.2	
25	2541431	5817124	3639414	554840.4	458451.6	883670.2	395679.1	660567.1	1587144	746379.4	652518.2	31771914	32168345	17935149
26	4755502	6787683	5261756	88283.39	1851.201		76219.33	1955.483	2645.813	140299.6		23093209	58027342	48171250
27	4967154	8722822	7400166	419812.4	68349.92						48893.04	4550516	13095224	4881891
28	894484.6	1340406	1053326	134004.6	454266.1		99384.26	366773.5		390341.3	327368.9	692084.8	1912928	1862687
29	104977.7	371570.3	93489.91								81672.94	384522.8	777549.6	
30	39028.16	167620.1	123493.4									693673.3	1894537	3190438
31	94526.52	169728.6	93021.65									388770.8	991671.6	805021.9
32	172382.6	281196	288127.2										404489.2	
33	4172.257	82409.17			1230.792							1124565	913830.2	431470.7
34	80916.46	232017.3	133376.7	26851.28					150155.8			286367.1	640805.2	175891.7
35	5151508	8141476	6638544	428557.3		135950.5	54486.56	139289.8		47475.13	40211.96	20003749	29952762	8167819
36	44807861	76839932	38077736	2842686	4612911	6879356	10567016	7690201	5439203	3429779	5393357	4.93E+08	5.33E+08	1.07E+08
37	65778152	1.28E+08	52616826	1940255	5255460	5582827	10723543	6521568	5051496	2643946	3777942	7.04E+08	7.93E+08	1.53E+08
38	2894742	9301388	4944327	56497.82					154053.6			20849694	46331696	17376674
39	241855.7	149141.3	134006.8	75096.55			224924.3			152503.2		436873.4	2868.042	188567.5
40	19041.77											581660.4		42866.47
41	66441.75	158685.2	92484.09									253536.8	246905.5	509967.6
42	9422599	16682974	17539364	336716.8	150917.2	27890.09	78009.42	98453.4	237148.9	105949.8	112853.8	15336028	21594980	13880151
43	502348.5	1195872	403120.9	34539.23		41306.59	42561.47	53009.41		32810.79	83247.57	117843.3	94647.96	
44	2699601	3865570	4359067	652977.3	891604.6	651162.3	1148457	878544.1	1863491	1084925	1177889	3167907	3878956	3417630
45	37466.24		44195.26										66829.09	
46													369614.8	511337.4
47	24398.64		73971.12										25298.33	45115.8
48	1663251	2614687	2244541	8188.035								7439843	8986782	2248495
49	60786.71	52243.18		293793	102128	30962.72	44509.38				53424.23	1499484	222515.2	1339418
50	410353.2	154013.1	113519.5	102745.1	83317.64		59249.49		102318.5		127667.8	1281438	514154.2	2407047
51	556658	672199.3	788093.8	391195.9			58876.56			55991.78		2017619	1433079	3184153
52		45919.09	36540.04									78660.25	64714.68	117408.3
53													90999.4	82914.51
54	4764663	3024078	2089588	463747.3	601975.1	288475.9	573755.2	198043.7	321089.9	112352	194634.1	5442688	1642768	5071369
55	2182643	1082354	961525.8	446779.5	442130.9	339684.7	477855.8	282595.5		151319.8	372778.2	2916378	693086.8	2991097
56	23994.31		70679.3									255574.3	476159.4	2295387
57	200813.1	361821.1	233848.4									800588.1	651701	684088.7
58	27233.99		17733.99									440204.4	66215.58	3146560
59	754466.6	1063060	1285435		94949.46				331502.3					
60	7087999	14060263	10915610	1723708								7928860	13566575	9246242
61							73471.9					197518.4	31616.58	192957.7

ein abundance, normalized

	ILT4	ILT5	ILT6	ILT7	F1	F2	F3	F4	F5	F6	F7	C1	C2	C3
1	1515216	426271.2	1303768		2208600	2178757		791465.5	1349258	860120				
2	931861.8	441344	748189.3	891085.4	1434580	1355717		711468.2	894672.7	793162.5				
3	4461625	2014158	3762655	5164837	7649357	7061666	190444.8	3724075	3971360	2872427	2019961	1		1
4	3644669	1680039	3505347	273421.2	5055683	4167383	90082.52	2180509	3455882	2633068				
5	1368718	469384.7	637284.8	247650.9	2140665	1732069	188609.6	1086685	1595693	1231098				
6	3113027	5141616	3947547	5412484	5726876	8172320	13914340	4923675	5749355	7677882	7727223	2	2	2
7	199129	236298.6	129839.4	250417.1	447607	563100		600725.7	620272.6	571093.2	189842.3			
8	90099044	98658215	1.11E+08	39607670	1.08E+08	1.27E+08	15107995	97349888	1.06E+08	1.18E+08	17816893	10	6	5
9		346297.2	91606.84		201564.3	916089.3		195264.3	845383.6	472934.5				
10			81107.04		179101.2	138129.2			89816.88	200440.1				
11	11136644	6500907	6273910	4362203	15855452	17926077	337882.9	7508431	8117596	7107725	648416.7			
12	1815486	1070386	1009324	594255.7	2355668	2657189		1002011	1511749	906368.5				
13	486983.8	264056.1			417028.2	725890.1								
14	2089588	1706152	1631462	423403.5	2411642	2980090	177133.8	939693.1	1622964	747027.4	128824.4		1	
15	1495831	1303039	953269.3	754588.2	1973101	2529847		1089758	1557513	748997.8				
16	14892216	11312693	9889322	10583170	16031951	17873653	843673.6	5752708	8173638	3320975	794691.7	2	1	2
17	270330.7	152811.3	438634	97003.03	146590.3	276809.4		108257	165580	178301.6				
18	13076938	6321925	6480164	2870014	36823846	38024670	116351.2	14069513	20077656	14658833	77310.24			
19	494758.1	38632.84	186584.5	229624	1789806	1398939		632928.2	869445.1	535487				
20	2736913	2049543	1303680	203865.3	4554758	4682177	103200	2948680	3711911	2472833	112958.9			
21	563549.7	477882.4	366281.9						153398	251666				
22	1096534	1093933	1563213	248747.3	2020591	2724256		1240447	1957202	2132463				
23	2400171	2716038	1964663	1383705	483873	670304.3	32639.16		234091.1	269900.4				
24	81983.68	48910.37	178350	108113.3	381531.2	390899.4		146713.1	364710.2	259520.2				
25	26489556	19324277	19814714	38446339	12129748	16491346	3152937	4940452	7830696	5698848	1441607	1	1	2
26	49400233	27355244	38572383	14766212	14895838	23829619	474197.4	9244529	9137210	8239223	229381.1	1		2
27	5722634	6875191	4603828	2856804	10462361	18116002	1965120	9655973	11742190	11587694	1090772	1		
28	1203225	707011.3	1106482	337693.8	2102930	2606363	294424.6	1738847	1804382	1649371	348175.7	1		1
29	710435.2	748105.9	935358.6	174676.5	410845.1	513034.3	309060.7	204072.9	500187.8	146393				
30	945765	437616.3	804316	1284606	604109.1	794018.9		75869.38	225641	193374.6				
31	518306.9	168743	804024.9	597413.6	344113	402444		183756.3	228479.5	145659.8				
32	48530.28	129675.9	294241.8	200657	519760.1	620969.2		335105.8	378530.7	451169.5				
33	866196.5	1435772	1401905	1505669	127668.3	256108	72245.84	8110.722	110934.7			1		
34	564015.4	534886	636528.4		213831.7	450945.3		157298.8	312329	208850.5	69765.97			
35	26956167	23704169	27907569	8385071	14821351	22395001	551121.3	10014352	10959612	10395094	1113493		1	1
36	4.86E+08	6.21E+08	2.95E+08	3.2E+08	1.32E+08	1.47E+08	8702569	87104914	1.03E+08	59624768	7385970	12	16	16
37	8.63E+08	9.13E+08	4.79E+08	3.8E+08	1.79E+08	2.31E+08	8532751	1.28E+08	1.72E+08	82391087	5041240	12	11	13
38	52966029	46430401	47023878	6635754	12653056	22954680	59880.93	5627277	12521023	7742171	146794.7			
39		1263.404	138111.4	132182.2	883367.1	134262.3	536677.7	470159.1	200766	209837.2	195118.6			1
40		589496.7	655593	166880.3	42082.78			37016.54						
41		132676.1	36651.3	960568.6	164909			129160.4	213613.3	144818				
42	12385102	17609259	10095980	20083603	23124540	34146870	1100468	18317202	22457713	27464356	874870	4	2	3
43	66215.3	62928.94	146633.7	490018.4	1385305	1576172		976547.9	1609818	631234.7	89741.09		1	1
44	2547067	3546284	3048184	3135300	6284308	7423538	1714007	5247930	5203621	6825731	1696589	2	2	3
45			51340.29		59929.19	83813.84		72833.06		69204.02				
46	180027.6	156519.4			43406.38	97797.45								
47	68004.09	31844.85	99273.62		173981.9	113738		47430.1		115829.1				
48	7744767	7589342	6169989	2618974	4020580	5041346	34306.36	3233302	3519749	3514659	21274.45			
49	1221156	656719.9	780215.3	3577029	322822.5	167206.2		118167.2	70326.93		763343.8	1	1	1
50	441146.8		544289.6	2225405	3046969	474772.8	137932.7	797712.3	207324.1	177756.7	266956	1		1
51	1153635	429948	474499	1950960	1876615	803451	442942.2	1082124	904878.2	1234052	1016419			1
52				348285.5	71178.05	47651.54			61813.78	57216.94				
53	43487.59			129641.6		320405.2								
54	1645096	553193.8	2895773	5134526	24477525	5554641	595568.9	9262338	4070850	3272022	1204925	4	3	4
55	466068.4	145274	1048327	5755041	14408348	2515729	381169.4	4242982	1457006	1505624	1160839	2	2	2
56	139906.2		14670.72	1623189	470712.8	460182.5		46644.11		110674.6				
57	853193.1	216639.3	497286.9	265351.9	1485949	757985.4		390373.7	487063.9	366176.1				
58	49966.37			639117.5	135304.6	66248.91		52941.93		27769.11				
59	1371358	964189.7	238950.1	512845.8	271945.9	1023977		1466657	1431033	2012824		1		
60	7986972	15308916	6653758	11469154	19932891	21259658	4167566	13778821	18927162	17092422	4478602			
61	41693.31	17293.33		485219.3	33164.44									1

	Protein abundance, count													
	C4	C5	C6	C7	ILT1	ILT2	ILT3	ILT4	ILT5	ILT6	ILT7	F1	F2	F3
1					1	2	1	2	1	2		2	2	
2					2	2	2	2	2	2	2	2	2	
3		2	1		4	7	7	7	6	7	6	6	6	1
4					3	10	7	10	8	10	3	11	11	1
5		1	1	1	1	4	1	6	3	3	2	7	8	1
6	2	1	1	2	10	18	14	13	17	17	10	17	17	6
7						1		1	1	1	1	1	1	
8	7	2	3	8	48	61	48	57	64	57	36	54	59	24
9						1			1	1		1	2	
10					1		1			1		1	1	
11	2		1	1	15	28	21	24	22	21	12	27	27	2
12					2	8	7	8	8	6	3	9	8	
13						1	1	3	2			2	3	
14					3	7	6	7	5	6	2	6	7	1
15					2	5	5	5	5	5	3	5	5	
16			3	1	8	16	15	15	14	15	11	16	16	4
17						2	1	2	2	2	1	1	2	
18	1				10	18	14	17	16	15	7	19	20	1
19					1	3	1	2	1	2	1	4	3	
20					2	2	2	2	2	2	1	2	2	1
21						1	1	1	1	1				
22					2	4	4	3	3	4	1	5	5	
23					2	4	4	4	5	4	4	2	4	1
24					1	1		1	1	1	1	2	2	
25	2	3	1	2	18	19	17	18	18	19	18	17	18	4
26	1	1	1		7	8	8	8	9	8	5	8	8	2
27				1	7	11	7	10	10	10	6	13	16	4
28	1		1	1	3	5	6	4	4	6	3	7	8	1
29				1	2	5		5	6	6	2	3	4	1
30					2	2	2	2	2	2	2	2	2	
31					1	2	2	2	1	1	1	1	1	
32						1		1	2	2	1	2	2	
33					4	3	3	5	6	6	5	1	2	1
34		1			1	3	1	2	3	4		1	4	
35	1		1	1	15	18	14	18	19	18	10	18	19	3
36	12	12	8	12	40	42	24	39	47	37	35	25	33	14
37	11	11	10	8	29	32	29	34	35	30	28	28	30	11
38		1			28	37	30	41	37	40	13	29	35	1
39			1		2	1	2		1	3	1	4	3	1
40					1		1		2	2	2	1		
41					1	1	2		1	1	2	1		
42	2	2	2	3	19	28	25	23	27	27	24	28	29	5
43	1		1	1	1	1		1	1	3	3	7	9	
44	3	2	3	2	6	8	8	8	8	9	9	10	12	4
45						1				1		1	1	
46						2	2	1	2			1	1	
47						1	1	1	1	1		1	2	
48					7	8	6	8	8	8	5	8	8	1
49				2	3	1	3	3	3	3	3	2	2	
50		1		1	7	5	8	4		5	6	10	5	1
51			1		5	6	7	8	3	5	5	7	4	1
52					1	1	1				1	1	1	
53						1	1	1			1		3	
54	1	3	2	1	4	4	4	4	3	4	4	5	4	3
55	2		1	1	3	2	4	2	2	3	4	4	3	1
56					2	4	4	2		1	4	3	4	
57					2	2	2	3	2	3	1	2	2	
58					2	1	4	1			2	2	1	
59		1						2	2	2	1	1	2	
60					7	9	8	10	10	9	9	10	10	7
61					2	1	2	1	1		2	1		

	F4	F5	F6	F7	C1	C2	C3	C4	C5	C6	C7	ILT1
1	1	2	1		Not Found	Not Found	Not Found	Not Found	Not Found	Not Found	Not Found	Peak Found
2	2	2	2		Not Found	Not Found	Not Found	Not Found	Not Found	Not Found	Not Found	High
3	6	6	6	1	Peak Found	Not Found	Peak Found	Not Found	Peak Found	Peak Found	Not Found	High
4	6	10	9		Not Found	Not Found	Not Found	Not Found	Not Found	Not Found	Not Found	High
5	4	6	6		Not Found	Not Found	Not Found	Not Found	Peak Found	Peak Found	Peak Found	Peak Found
6	16	17	17	9	Peak Found	Peak Found	Peak Found	Peak Found	Peak Found	Peak Found	Peak Found	High
7	1	1	1	1	Not Found	Not Found	Not Found	Not Found	Not Found	Not Found	Not Found	Not Found
8	57	60	59	35	Peak Found	Peak Found	Peak Found	High	Peak Found	Peak Found	Peak Found	High
9	1	1	1		Not Found	Not Found	Not Found	Not Found	Not Found	Not Found	Not Found	Not Found
10		1	1		Not Found	Not Found	Not Found	Not Found	Not Found	Not Found	Not Found	Peak Found
11	19	23	23	5	Not Found	Not Found	Not Found	Peak Found	Not Found	Peak Found	Peak Found	High
12	6	8	6		Not Found	Not Found	Not Found	Not Found	Not Found	Not Found	Not Found	Peak Found
13					Not Found	Not Found	Not Found	Not Found	Not Found	Not Found	Not Found	High
14	3	4	2	1	Not Found	Peak Found	Not Found	Not Found	Not Found	Not Found	Not Found	High
15	5	5	4		Not Found	Not Found	Not Found	Not Found	Not Found	Not Found	Not Found	High
16	13	16	11	3	Peak Found	Peak Found	Peak Found	Not Found	Not Found	Peak Found	Peak Found	High
17	1	1	1		Not Found	Not Found	Not Found	Not Found	Not Found	Not Found	Not Found	Not Found
18	15	20	14	2	Not Found	Not Found	Not Found	Peak Found	Not Found	Not Found	Not Found	High
19	3	3	3		Not Found	Not Found	Not Found	Not Found	Not Found	Not Found	Not Found	Peak Found
20	2	2	2	1	Not Found	Not Found	Not Found	Not Found	Not Found	Not Found	Not Found	High
21		1	1		Not Found	Not Found	Not Found	Not Found	Not Found	Not Found	Not Found	Not Found
22	4	5	5		Not Found	Not Found	Not Found	Not Found	Not Found	Not Found	Not Found	High
23		2	2		Not Found	Not Found	Not Found	Not Found	Not Found	Not Found	Not Found	High
24	1	2	2		Not Found	Not Found	Not Found	Not Found	Not Found	Not Found	Not Found	Peak Found
25	10	13	13	7	Peak Found	Peak Found	Peak Found	Peak Found	Peak Found	Peak Found	Peak Found	High
26	5	6	7	4	Peak Found	Not Found	Peak Found	Peak Found	Peak Found	Peak Found	Not Found	High
27	9	12	12	3	Peak Found	Not Found	Not Found	Not Found	Not Found	Not Found	Peak Found	High
28	6	7	7	1	Peak Found	Not Found	Peak Found	Peak Found	Not Found	Peak Found	Peak Found	High
29	1	3	2		Not Found	Not Found	Not Found	Not Found	Not Found	Not Found	Peak Found	High
30	1	2	2		Not Found	Not Found	Not Found	Not Found	Not Found	Not Found	Not Found	High
31	1	1	1		Not Found	Not Found	Not Found	Not Found	Not Found	Not Found	Not Found	Peak Found
32	1	1	1		Not Found	Not Found	Not Found	Not Found	Not Found	Not Found	Not Found	Not Found
33	1	2			Peak Found	Not Found	Not Found	Not Found	Not Found	Not Found	Not Found	High
34	1	3	2	1	Not Found	Not Found	Not Found	Not Found	Peak Found	Not Found	Not Found	High
35	15	17	19	7	Not Found	Peak Found	Peak Found	Peak Found	Not Found	Peak Found	Peak Found	High
36	23	31	25	13	High	High	High	High	High	High	High	High
37	24	29	28	11	High	High	High	High	High	High	High	High
38	15	27	23	2	Not Found	Not Found	Not Found	Not Found	Peak Found	Not Found	Not Found	High
39	4	3	3	1	Not Found	Not Found	Peak Found	Not Found	Not Found	Peak Found	Not Found	High
40	1				Not Found	Not Found	Not Found	Not Found	Not Found	Not Found	Not Found	High
41	1	2	1		Not Found	Not Found	Not Found	Not Found	Not Found	Not Found	Not Found	Peak Found
42	24	26	28	6	Peak Found	High	Peak Found	High	Peak Found	Peak Found	High	High
43	4	9	4	1	Not Found	Peak Found	Peak Found	Peak Found	Not Found	Peak Found	Peak Found	Peak Found
44	9	10	10	4	Peak Found	Peak Found	Peak Found	Peak Found	Peak Found	Peak Found	Peak Found	High
45	1		1		Not Found	Not Found	Not Found	Not Found	Not Found	Not Found	Not Found	Not Found
46					Not Found	Not Found	Not Found	Not Found	Not Found	Not Found	Not Found	Not Found
47	1		1		Not Found	Not Found	Not Found	Not Found	Not Found	Not Found	Not Found	Not Found
48	6	7	8	1	Not Found	Not Found	Not Found	Not Found	Not Found	Not Found	Not Found	High
49	1	1		3	High	Peak Found	High	Not Found	Not Found	Not Found	Peak Found	High
50	7	4	3	1	Peak Found	Not Found	Peak Found	Not Found	Peak Found	Not Found	Peak Found	High
51	3	4	3	3	Not Found	Not Found	Peak Found	Not Found	Not Found	Peak Found	Not Found	High
52		1	1		Not Found	Not Found	Not Found	Not Found	Not Found	Not Found	Not Found	Peak Found
53					Not Found	Not Found	Not Found	Not Found	Not Found	Not Found	Not Found	Not Found
54	4	4	4	4	High	High	High	High	Peak Found	High	High	High
55	3	3	3	3	Peak Found	Peak Found	Peak Found	Peak Found	Not Found	Peak Found	Peak Found	High
56	1		2		Not Found	Not Found	Not Found	Not Found	Not Found	Not Found	Not Found	Peak Found
57	2	2	2		Not Found	Not Found	Not Found	Not Found	Not Found	Not Found	Not Found	Peak Found
58	1		1		Not Found	Not Found	Not Found	Not Found	Not Found	Not Found	Not Found	Peak Found
59	2	2	2		Peak Found	Not Found	Not Found	Not Found	Peak Found	Not Found	Not Found	Not Found
60	10	10	10	8	Not Found	Not Found	Not Found	Not Found	Not Found	Not Found	Not Found	High
61					Not Found	Not Found	Peak Found	Not Found	Not Found	Not Found	Not Found	High

	Protein found in file										
	ILT2	ILT3	ILT4	ILT5	ILT6	ILT7	F1	F2	F3	F4	F5
1	High	High	High	High	High	Not Found	High	High	Not Found	High	High
2	High	High	High	High	High	High	High	High	Not Found	High	High
3	High	High	High	High	High	High	High	High	Peak Found	High	High
4	High	High	High	High	High	High	High	High	Peak Found	High	High
5	High	Peak Found	High	High	High	High	High	High	Peak Found	Peak Found	High
6	High	High	High	High	High	High	High	High	High	High	High
7	High	Not Found	High	High	High	High	High	High	Not Found	High	High
8	High	High	High	High	High	High	High	High	High	High	High
9	High	Not Found	Not Found	High	High	Not Found	High	High	Not Found	High	High
10	Not Found	Peak Found	High	High	High	Not Found	High	High	Not Found	High	Peak Found
11	High	High	High	High	High	High	High	High	Peak Found	High	High
12	High	High	High	High	High	High	High	High	Not Found	High	High
13	High	High	High	High	High	Not Found	High	High	Not Found	High	High
14	High	High	High	High	High	High	High	High	Peak Found	High	High
15	High	High	High	High	High	High	High	High	Not Found	High	High
16	High	High	High	High	High	High	High	High	Peak Found	High	High
17	Peak Found	Peak Found	Peak Found	Peak Found	High	Peak Found	High	High	Not Found	Peak Found	High
18	High	High	High	High	High	High	High	High	Peak Found	High	High
19	High	High	High	High	High	High	High	High	Not Found	High	High
20	High	High	High	High	High	High	High	High	Peak Found	High	High
21	High	High	High	High	High	Not Found	Not Found	Not Found	Not Found	Not Found	Peak Found
22	High	High	High	High	High	High	High	High	Not Found	High	High
23	High	High	High	High	High	Peak Found	High	High	Peak Found	Not Found	High
24	Peak Found	Not Found	Peak Found	Peak Found	Peak Found	Peak Found	High	High	Not Found	High	High
25	High	High	High	High	High	High	High	High	High	High	High
26	High	High	High	High	High	High	High	High	Peak Found	High	High
27	High	High	High	High	High	High	High	High	High	High	High
28	High	High	High	High	High	Peak Found	High	High	Peak Found	High	High
29	High	Not Found	High	High	High	Peak Found	Peak Found	High	Peak Found	Peak Found	High
30	High	High	High	High	High	High	High	High	Not Found	Peak Found	High
31	Peak Found	Peak Found	High	Peak Found	High	Peak Found	Peak Found	Peak Found	Not Found	Peak Found	Peak Found
32	High	High	High	High	High	Peak Found	Peak Found	High	Not Found	Peak Found	High
33	High	High	High	High	High	High	Peak Found	High	Peak Found	Peak Found	Peak Found
34	High	Peak Found	High	High	High	Not Found	High	High	Not Found	High	High
35	High	High	High	High	High	High	High	High	Peak Found	High	High
36	High	High	High	High	High	High	High	High	High	High	High
37	High	High	High	High	High	High	High	High	High	High	High
38	High	High	High	High	High	High	High	High	Peak Found	High	High
39	Peak Found	Peak Found	Not Found	High	High	Peak Found	High	Peak Found	Peak Found	High	High
40	Not Found	Peak Found	Not Found	High	High	High	Peak Found	Not Found	Not Found	Peak Found	Not Found
41	Peak Found	High	Not Found	Peak Found	Peak Found	High	Peak Found	Not Found	Not Found	Peak Found	Peak Found
42	High	High	High	High	High	High	High	High	High	High	High
43	High	Not Found	Peak Found	Peak Found	Peak Found	Peak Found	High	High	Not Found	High	High
44	High	High	High	High	High	High	High	High	High	High	High
45	Peak Found	Not Found	Not Found	Not Found	Peak Found	Not Found	High	High	Not Found	Peak Found	Not Found
46	High	High	High	Peak Found	Not Found	High	Peak Found	High	Not Found	Not Found	Not Found
47	Peak Found	High	Peak Found	Peak Found	High	High	High	Peak Found	Not Found	Peak Found	Not Found
48	High	High	High	High	High	High	High	High	Peak Found	High	High
49	High	High	High	High	High	High	High	High	High	High	Peak Found
50	High	High	High	Not Found	High	High	High	High	Peak Found	High	High
51	High	High	High	Peak Found	High	High	High	High	Peak Found	Peak Found	Peak Found
52	Peak Found	Peak Found	Not Found	Not Found	Not Found	High	High	High	Not Found	Not Found	Peak Found
53	High	High	Peak Found	Not Found	Not Found	High	High	High	Not Found	Not Found	Not Found
54	High	High	High	High	High	High	High	High	Peak Found	High	High
55	High	High	Peak Found	High	High	High	High	High	Peak Found	High	High
56	High	High	Peak Found	Not Found	Peak Found	High	High	High	Not Found	Peak Found	Not Found
57	High	High	High	High	High	High	High	High	Not Found	High	High
58	Peak Found	High	Peak Found	Not Found	Not Found	High	Peak Found	Peak Found	Not Found	Peak Found	Not Found
59	High	Not Found	High	High	High	High	High	High	Not Found	High	High
60	High	High	High	High	High	High	High	High	High	High	High
61	Peak Found	Peak Found	Peak Found	Peak Found	Not Found	High	Peak Found	Not Found	Not Found	Not Found	Not Found

	F6	F7
1	High	Not Found
2	High	Not Found
3	High	Peak Found
4	High	Not Found
5	High	Not Found
6	High	High
7	High	Peak Found
8	High	High
9	High	Not Found
10	High	Not Found
11	High	Peak Found
12	High	Not Found
13	High	Not Found
14	High	Peak Found
15	High	Not Found
16	High	Peak Found
17	High	Not Found
18	High	Peak Found
19	High	Not Found
20	High	Peak Found
21	Peak Found	Not Found
22	High	Not Found
23	High	Not Found
24	High	Not Found
25	High	High
26	High	Peak Found
27	High	High
28	High	Peak Found
29	High	Not Found
30	High	Not Found
31	Peak Found	Not Found
32	High	Not Found
33	Not Found	Not Found
34	High	Peak Found
35	High	Peak Found
36	High	High
37	High	High
38	High	Peak Found
39	High	Peak Found
40	Not Found	Not Found
41	Peak Found	Not Found
42	High	High
43	High	Peak Found
44	High	High
45	High	Not Found
46	High	Not Found
47	High	Not Found
48	High	High
49	Not Found	High
50	High	Peak Found
51	Peak Found	Peak Found
52	High	Not Found
53	Not Found	Not Found
54	High	High
55	High	High
56	High	Not Found
57	High	Not Found
58	Peak Found	Not Found
59	High	Not Found
60	High	High
61	Not Found	Not Found
